# From Grouping to Coupling: A New Perceptual Organization in Vision, Psychology, and Biology

**DOI:** 10.3389/fpsyg.2016.01051

**Published:** 2016-07-14

**Authors:** Baingio Pinna, Daniele Porcheddu, Katia Deiana

**Affiliations:** ^1^Department of Humanities and Social Sciences, University of SassariSassari, Italy; ^2^Department of Economics and Business, University of SassariSassari, Italy

**Keywords:** perceptual organization, perceptual coupling, shape perception, perceptual grouping, Gestalt psychology, visual illusions

## Abstract

In this work, perceptual organization has been studied with the same spirit and phenomenological methods used by Gestalt psychologists. This was accomplished through new conditions that cannot be explained in terms of the classical principles of grouping. Perceptual grouping represents the way through which our visual system builds integrated elements on the basis of the maximal homogeneity among the components of the stimulus pattern. Our results demonstrated the inconsistency of organization by grouping, and more importantly, the inconsistency of the principle of similarity. On the contrary, they suggested the unique role played by the principle of dissimilarity among elements that behaves like an accent or a visual emphasis within a whole. The principle of accentuation was here considered as imparting a directional structure to the elements and to the whole object thus creating new phenomena. The salience of the resulting phenomena reveals the supremacy of dissimilarity in relation to similarity and the fact that it belongs to a further organization dynamics that we called “coupling.” In biology, coupling and its principle of accentuation are very strongly related to disruptive camouflage. Moreover, they are source of sexual attraction. They advertise the presence and elicit species identification/communication. In human beings accentuation is needed to show ourselves to others, to understand the way we dress, choose, and create clothes or invent fashion, the way we change our body accentuating several parts and hiding some others, the way we use maquillage. The existence of maquillage itself is derived from the need to accentuate something with the purpose to increase sexual attraction, to exhibit physical strength and beauty, to show or hide social status (e.g., being the king, a warrior, a priest, etc.). Last but not least, accentuation plays a basic role also in making it easier or difficult to read and understand written words.

## Introduction

**Figure 1:**
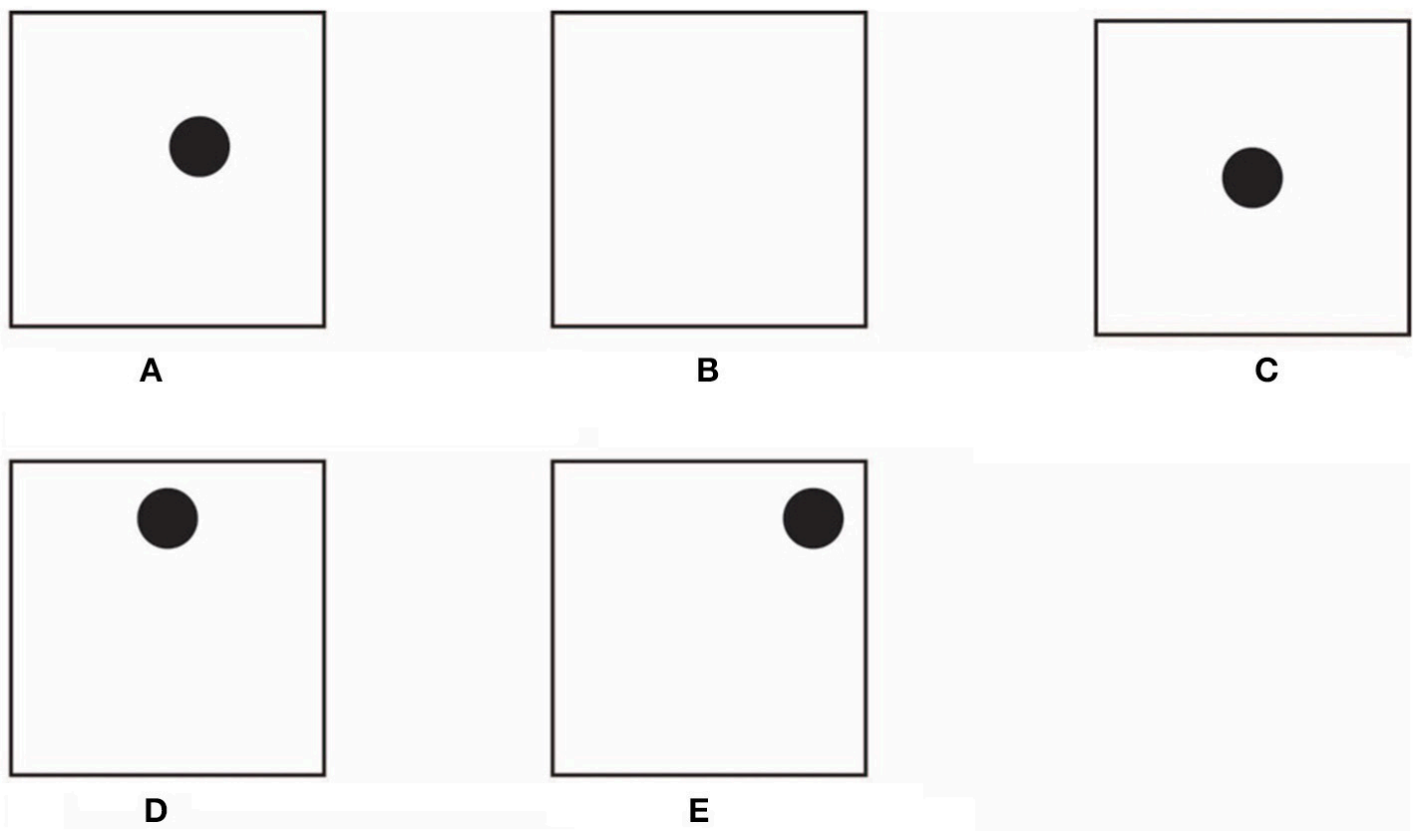
**The role of the center of a figure in Arnheim's theory (A–C)**. The disk heightens and accentuates different components of the square **(D–E)**.

According to Arnheim (1954, 1974, 1982) the center is a special location within a figure. When a disk is positioned slightly away from the center of a square, the resulting asymmetry is perceived as a “tension” that strives the disk toward the center (Figure 1A). Moreover, there is no need to measure it in order to perceive at a glance that the disk lies off-center.

Arnheim suggested that the center behaves like the origin of forces. They are considered like vectors radially oriented, which emanating from it, create a force field and a source of energy. According to the Gestalt tradition, the use of terms such as “forces” in Arnheim (1954, 1974, 1982) is taken in a fairly literal sense. They are assumed to be real in both psychological and physical domains (Arnheim, 1971, 1974).

Phenomenally, it is worthwhile to add that the center, clearly visible in Figure 1A, is more undetermined when the inner disk of Figure 1A is removed (Figure 1B). This suggests that if the center shows the outlying position of the circle due to its force field, the opposite is also true, thus, the center is highlighted by the presence and position of the disk. The disk does not behave only like a test body within the force field of the center of mass, but it rather appears to highlight the presence and the location of the center. On that basis, in Arnheim's terms, the disk should have its own force field interacting with the one of the center, however this is not precisely what Arnheim stated.

Phenomenally, the mutual definition and visual accentuation of the center and of the disk can be indirectly supported by the fact that the term “center” was spontaneously reported by the subjects (see Section Materials and Methods) when they described Figure 1A (“a square with a circle lying off-center”), while the center was not mentioned at all when the task was to describe Figure 1B (just “a square”). Linguistically, the center of Figure 1B is phenomenally implicit (see Pinna et al., 2015).

When the disk is placed in the center (Figure 1C) the descriptions made the center explicit again (“a disk placed in the center of the square”). However, when the disk is shifted further, as illustrated in Figures 1D,E, the center, again, is not mentioned at all and the new reference becomes the upper side (Figure 1D, “the disk is near the upper side of the square”) and the right upper angle (Figure 1E, “the disk is near the right upper angle”). These simple and spontaneous descriptions suggest an alternate way to explain a large set of effects (see next sections) if we assume that a description traces the structure of what is perceived.

These outcomes, apparently trivial, suggest that the disk operates more actively than what is expected to do on the basis of Arnheim's theory. In fact, the disk seems to heighten and accentuate different components of the square. Without invoking force fields, a simpler idea suggests that, since sides and angles, together with the center, are all basic components of the square, as such, they could be highlighted by elements such as disks, for example, that are related or coupled with that specific attribute.

On the contrary, according to Arnheim's theory the disk is unilaterally influenced, mostly by the center of the square and more weakly by the cross-shaped framework of the central vertical and horizontal axes and by the diagonals. Indeed, according to Arnheim, the center is considered as the main locus of attraction and repulsion and it defines itself through the crossing of the four main diagonals of the square. The other components within the square, sides and angles, are less powerful than the center. This theory is in line with the Gestalt approach of perceptual organization, mainly based on the grouping and ungrouping of elements and conceived on the basis of principles that define the maximal homogeneity among them (Musatti, 1931; Kanizsa, 1979, 1980). Therefore, the disk and the square are considered and perceived as belonging to two distinct groups reciprocally segregated on the basis of their dissimilarity.

It follows that the disk can, at most, be considered like a test body for the force field generated by the center of mass of the square acting as an attractor. More generally, the resulting Gestalt, given the available stimulation, emerges from the global force field and elicits the simplest possible organization, or minimum solution.

**Figure 2:**
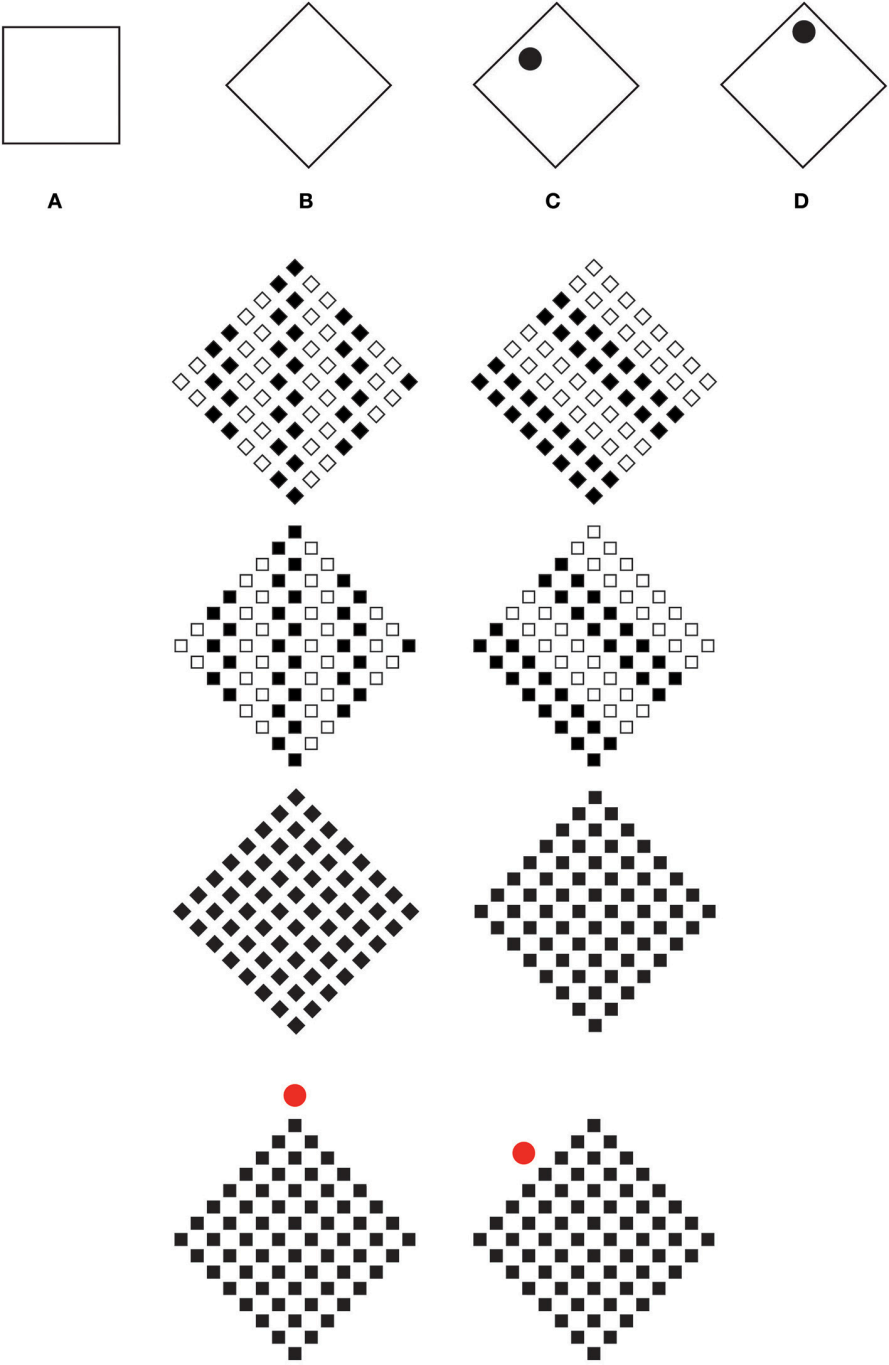
**Mach's square/diamond illusion (A–B)**. “A rotated square” **(C)** and “a diamond” **(D)**.

Related to the components of the square, sides and angles, and, hence, to the conditions illustrated in Figures 1D,E, is Mach's square/diamond illusion (Schumann, 1900; Mach, 1914/1959) demonstrating that the same geometrical figure is perceived like a square when its sides are vertically and horizontally oriented, or like a diamond when they are oblique (Figures 2A,B). Moreover, the diamond appears larger than the square. Schumann (1900) suggested that, under these conditions, visual attention is placed on the vertical-horizontal axes, which in the diamond condition are clearly longer. His explanation is supported by the results of a simple control experiment according to which, by focusing our attention on one side of the diamond rather than on one angle, during the comparison of Figures 2A,B, the apparent size difference between the square and the diamond is clearly reduced or even annulled.

From a phenomenal point of view, the square and the diamond of Figures 2A,B are different since they highlight two different components of the geometrical square. As the square shapes of Figures 2A,B are both made up of sides and angles, they should show both phenomenal properties such as “sidedness,” i.e., the appearance of being flat and stable, and “pointedness,” i.e., the property of being sharp and unstable. However, within a square and a diamond, these two attributes are only apparently equipollent and symmetrical in strength. In reality, within the square, sidedness is much stronger than pointedness, while within the diamond pointedness is shown more strongly.The perceived strength of one or the other property is influenced by the vertical/horizontal and gravitational axis that, although invisible, plays like a reference frame accentuating sidedness against pointedness, in the square, and pointedness against sidedness, in the diamond. Consequently, the square is perceived when the sides are vertical and horizontal, while a diamond is seen when its angles are vertical and horizontal. As such, the vertical-horizontal organization favors the emergence of the sides, in the case of the square, and of the angles/vertices, in the case of the diamond (Pinna, 2010a,b, 2012a, 2015; Pinna and Albertazzi, 2011). Moreover, given that the square is made up of sides and angles, then, it is expected to reveal phenomenal attributes related to them, i.e., “sidedness” or “pointedness,” attributes that can be highlighted or accentuated if the disk of Figure 1A plays as such.

Figures 1D,E already proved it indirectly through the spontaneous descriptions of our subjects. A more effective demonstration, however, can be obtained by using the diamond of Figure 2B, as shown in Figures 2C,D. Under these conditions, the geometrical diamond of Figure 2C is perceived as a rotated square, while the same geometrical diamond of Figure 2D is seen uniquely as a diamond. It is worth defining that phenomenally a rotated square and a diamond are two different shapes, not only because they have two different names, but, mostly, because they reveal different phenomenal attributes, i.e., pointedness, in the case of diamonds, and sidedness, in the case of rotated squares.

These results can be generalized as follows: all else being equal, the perceived shape switches from one shape to another by means of the accentuation of the sidedness or of the pointedness caused by the position of the disk. This occurs independently from the vertical/horizontal axes (see also Pinna, 2010a,b; Pinna and Albertazzi, 2011).

More in detail, by comparing Figures 1D,E with Figures 2C,D, the strength of accentuation due to the disk appears asymmetric. In fact, in the geometrical square conditions (Figures 1D,E), sidedness appears stronger than pointedness, therefore, the effect of accentuation and its power of switching from one shape to another, is weaker than the one observed in the geometrical diamond condition (Figures 2C,D), where, on the contrary, the figure is more easily switchable from diamond to rotated square.

At first glance, the term “accentuation” might look similar to the more general notion of “salience.” Before proceeding, it is useful to clarify how accentuation differs from visual salience and under which circumstances accentuation and salience diverge. These points will be reconsidered more in details in the next Sections (Accentuation vs. Salience and Coupling vs. Medium-Range Grouping, Accentuations in Biology, and Accentuation in Humans), when the principle of accentuation will be phenomenologically deepened.

Visual salience is a distinct subjective perceptual quality making some items pop out from their neighbors and immediately grab attention (Itti, 2007). This is the case of the black circles placed inside the diamonds of Figures 2C,D. As visually salient stimuli, they immediately attract our attention. This implies that the core of visual salience is mainly (but not only, see Desimone and Duncan, 1995; Itti and Koch, 2001; Navalpakkam and Itti, 2007) a bottom-up, stimulus-driven, signal stating that a specific element is sufficiently different from its surroundings to attract and be worthy for attention (see Itti, 2007). As such, visual salience inspired a large number of experiments mainly concerned with tasks, such as looking for an odd-man-out target embedded within an array of distractors. This core is not changed by recent works demonstrating that top-down or task-based modulation can influence visual salience (e.g., Yeshurun and Carrasco, 1998; Navalpakkam and Itti, 2007).

The kind of accentuation and accents studied in this work suggest that something emphasizes something else. Something that emerges, due to its salience accentuates a property of another object, of a different separated object. It is not the emergence of the accent itself, i.e., of the black circle of Figure 2D that matters, but the properties of something else, the sidendness and pointedness of the diamond. The target of the accentuation is not the circle but the diamond that, due to the accentuation can appear as a rotated square. In this sense, accentuation is not intended as the prominence of something relative to the normal, e.g., a condition where there is an accentuation of male or female characteristics in biology. This is salience not accentuation. Accentuation is a sign that highlights not itself but something else nearby.

The second row of Figure 2 shows some examples useful to demonstrate the necessary distinction between salience and accentuation. Here, visual salience is the distinct subjective perceptual quality of vertical and oblique grouping of small elements that immediately grab visual attention. Accentuation starts now. As a matter of fact, the salience of the two directions accentuates the local and global directions of the small and large squares, therefore the single diamonds are pereived as diamonds (second row-left) and as rotated squares (second row-right). The same kinds of accentuations occur also for the large squares made of small squares. In the third row of Figure 2, the small diamonds are replaced with small squares. As a consequence, although the salient directions are the same (i.e., vertical and oblique), the accentuated shapes are squares (left) and diamonds (right). In the third row of Figure 2, two controls are shown. Finally, in the fourth row of Figure 2, the same results of the third row can be accentuated by a red circle. The salience of each red circle is different from the complex long range effects due to the accentuation induced by it. It is important to underline that these figures have not been specified by letters, since each letter can accentuate or interfere in the accentuation process, thus changing the final result (see the first row of Figure 2; see also Pinna and Sirigu, 2011). This fact advocates the distinction between salience and accentuation. In fact, although the letters do not manifest any saliency quality, they can accentuate the result in favor or against the result obtained by the other accents. Examples in everyday life demonstrate that visual effect can be accentuated by something that is not necessarily salient.

On the basis of these phenomenal results and theoretical arguments, the following questions emerge. Since these effects are the result of perceptual organization, can they be explained in terms of perceptual grouping only? or a new kind of organization should be introduced? Can accentuation imparted by the disk be interpreted as a special case of visual attention? Under which conditions and according to which attribute variations does accentuation occur, increase or decrease? And more, is the phenomenon due to the interaction between the accentuating element and the one accentuated unilaterally? or are they reciprocally responsible of accentuating one another? Which are the main effects of coupling in biology?

## Materials and methods

### Subjects

The experiments were carried out with different groups of 14 undergraduate students. Subjects had only very little and basic knowledge of Gestalt psychology and visual illusions, and they were absolutely unaware both to the stimuli and to the purpose of the experiments. They were mixed groups of both male and female subjects with normal or corrected-to-normal vision.

### Stimuli

The stimuli used were all the figures described and shown in the next sections. The overall sizes of the visual stimuli were ~3.5° visual angle. The figures presented were shown on a computer screen with ambient illumination from a Osram Daylight fluorescent light (250 lux, 5600°K). Stimuli were also displayed on a 33 cm color CRT monitor (Sony GDM-F520 1600 × 1200 pixels, refresh rate 100 Hz), driven by a MacBook Pro computer with an NVIDIA GeForce 8600M GT. As far as viewing is concerned, it was binocular in the frontoparallel plane at a distance of 50 cm from the monitor.

### Procedure

The procedure used consists of two methods highly related to those also employed by Gestalt psychologists.

#### Phenomenological task

In this task subjects were asked to report spontaneously what they perceived by giving a complete description of the main visual property. The descriptions were provided by at least 10 out of 14 subjects and were reported concisely within the main text to aid the reader in the stream of argumentations. All the descriptions were evaluated by three graduate students of linguistics, totally naive as to the hypotheses, in order to get a fair representation of the descriptions which were given by the observers.

During their task, subjects could make free comparisons, lead to confrontations, add comments as afterthought, see in different ways, at different distance, etc.; but they could also match the stimulus with every other one they considered appropriate. All variations and possible comparisons arising during the free exploration were written down by the experimenter. The selection of the stimuli, with opposite conditions and controls, and the possible comparisons among the stimuli, were strategically combined so as to avoid the problem of generating biased experiences. This intent was fully achieved as it can be clearly seen by the differences shown in the results (see next sections).

#### Scaling task

With this task subjects were expected to rate (in percent) the descriptions of the specific attribute obtained in the phenomenological experiments. At this stage, new groups of 12 subjects were asked to scale the relative strength or salience (in percent) of the descriptions of the phenomenological task. In fact, they were asked: “please rate whether this statement is an accurate reflection of your perception of the stimulus, on a scale from 100 (perfect agreement) to 0 (complete disagreement).” Throughout the whole text, we reported descriptions, whose mean ratings were >80. For deeper insights into these tasks and procedure (see Pinna and Reeves, 2009; Pinna, 2010a,b; Pinna and Albertazzi, 2011; Pinna and Sirigu, 2011, 2016).

#### Magnitude estimation

The conditions illustrated in **Figures 6–10**, crucial for our purposes, were compared, and rated through the method of magnitude estimation. In this method, observers were presented a series of stimuli and they were instructed to scale the relative strength (in percent, where 100 is the maximal strength and 0 the minimal) of the attribute perceived (see the next sections for each stimulus and atttribute condition). The main advantage of this method is that observers can make full comparisons between all the stimuli, when presented all at once, thus minimizing the task complexity based on memory load. The stimuli order was fully randomized.

## Preliminary results: different kinds of perceptual organization?

### The Gestalt approach to grouping

**Figure 3:**
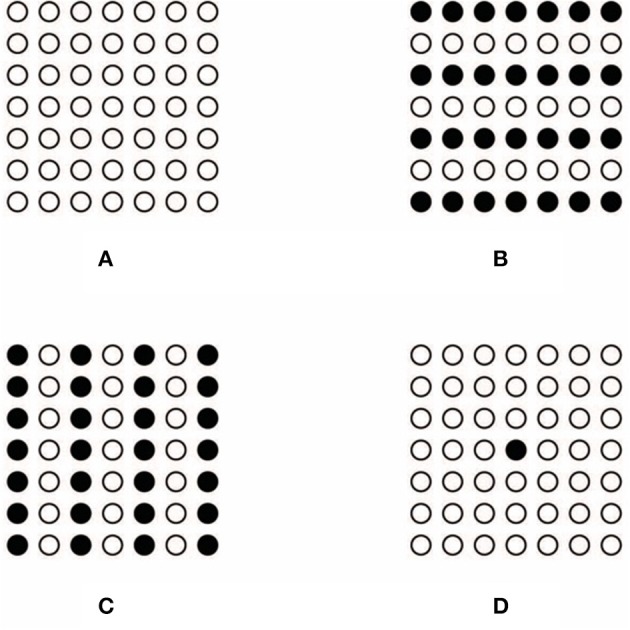
**Conditions showing the Gestalt grouping principle of similarity**. Small squares group together in a large square shape **(A)**. Similarity is responsible for grouping element components in rows **(B)**. Similarity is responsible for grouping element components in columns **(C)**. Only one circle is segregated from the others **(D)**.

According to Wertheimer (1923) elements, like the small empty circles illustrated in Figure 3A, group together in a large square-shape segregated from the background according to general grouping principles that under these conditions are synergistically related. These principles are: proximity, similarity, good continuation, closure, symmetry, convexity, Prägnanz, and parallelism.

In Figures 3B,C, the principle of similarity is responsible for grouping the element components in rows and columns. We suggest that similarity plays the main role also in Figure 3D, where only one circle appears segregated from the others. In fact, the empty circles group together as a uniform pattern from where the singularity of the dissimilar element segregates and pops out as a figure. The empty circles ungroup the only dissimilar circle. More in details, the empty circles group on the basis of the similarity and, at the same time, the filled circle is ungrouped from the others due to its dissimilarity. This suggests that similarity and dissimilarity, segregation and unification, grouping, and ungrouping are here considered as being two sides of the same coin. There is no antinomy between them, differently from what was suggested by Vicario (see Vicario, 1975, 1998; Luchins and Luchins, 1998).

In addition, the only filled circle, although different from the others, is geometrically similar to them since it is included like a knot in the squared lattice/grid made up of the totality of elements. Moreover, although it is dissimilar in one sense, due to its color, it is also similar to the other circles according to further similarity attributes, as shape, spatial location and common distance. Furthermore, the clear belongingness of the filled circle to the whole squared lattice is strongly demonstrated also by the fact that it reveals and highlights the center of the lattice. As a matter of fact, phenomenally, in spite of its dissimilarity, the filled circle is not segregated as something else but it appears as being the center of the lattice. As a result, its dissimilarity and its central position makes it special. As spontaneously reported by our subjects: “the circle is different because it is and it represents the center of the whole figure,” Consequently, its dissimilarity highlights and accentuates the center and, at the same time, its central position accentuates its dissimilarity. It follows the phenomenal result according to which, the highlighted center, due to the dissimilar element, appears more salient than the one perceived in Figure 1A, where the search for the exact position of the center requires an amount of time significantly higher than the one required to perceive the center of Figure 1D.

In summary, the principle of similarity reveals the uniformity of the surrounding empty circles and it segregates the uniqueness and dissimilarity of the filled circle. Besides, the play of similarities/dissimilarities is even more useful to highlight the center and to reinforce the wholeness of the squared lattice made up of circles that, under this condition appears more stable and static.

### Limits of the Gestalt approach

**Figure 4:**
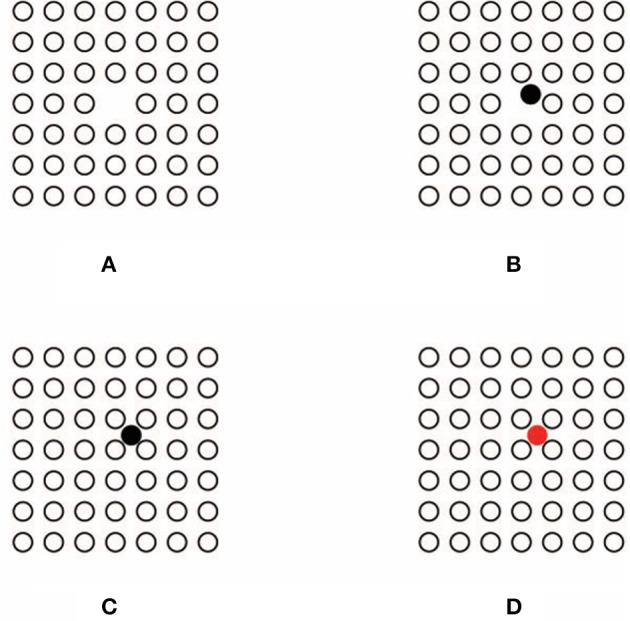
**The perception of an absence (A), of a wrong spatial position (B), of an intruder (C–D)**.

When the dissimilar element is removed (Figure 4A), the “absence of a circle” is clearly perceived by our subjects (an illusory square touching two lateral and up/down circles can also be perceived although none of our subjects reported this outcome). This result is paradoxical since the change in the spatial distances among the circles strongly reveals the “presence” of a central circle due to its “absence.” Indeed, the center appears as “a missing circle” and the whole pattern “as having a circle that is absent.”

This paradox is more linguistic than perceptual. In fact, the previous words, although linguistically antinomic are not antinomic within the perceptual domain. In other words, the perception of “a missing element” is a common experience, although it can be logically antinomic. Nevertheless, this is a complex result that requires to be adequately explained possibly through the similarity among the elements, without invoking the more ambiguous Prägnanz principle. In short, among the texturized similarities of empty circles, the missing one creates a change and a gap, and hence, a dissimilarity in the net of distances that, as such, behaves in the same way as the net of circles of Figure 3D. As a consequence, dissimilarity becomes “something” that appears like “nothing” or like an “absence” (as clearly reported by subjects). Briefly, the absence is highlighted by the broken similarity and uniformity of distances among the circles and, at the same time, it accentuates the uniformity and fullness of the lattice. As in Figure 3D, the center pops up due to the absence that is not an empty space but “something” belonging to the lattice of elements. In this way, the absence here appears as phenomenally different from what is perceived as an empty space among elements, namely, the interspace surrounding the circles. In fact, while the interspace is defined by similarity, the absence is defined by dissimilarity. To sum up, similarity can be considered like a principle of full homogeneity that highlights changes and dissimilarities within its inner homogeneity.

Although the absence can be considered as derived from the similarity principle, this is not the right acceptation that could be ascribed by Gestalt psychologists. The principle of similarity was, at first, intended to determine the grouping *per-se* without any other phenomenological meaning, as for example the possibility to induce emergent attributes like “absences.” The role of similarity and, more generally, of the Gestalt principles of grouping is to rule “what is or stays with what,” i.e., the phenomenon of grouping and not absences or other kinds of singularities (Wagemans et al., 2012a,b). Therefore, under this acceptation, the expected result for Figure 4A is a group of empty circles *tout court*.

The limited predictions of the Gestalt principles are more distinctly shown in Figure 4B. Now the filled circle of Figure 3D is perceived shifted from the center and it appears as placed in the “wrong” spatial position. Besides, the center of the whole pattern appears like an attractor for the wrong circle, whose replacement at the center would become the solution for the involved dissimilarities. Once more, the filled circle reveals the center of the whole pattern for the fact of its being “wrong” and, at the same time, the center of the pattern highlights the wrong place of the circle. In terms of the Gestalt principle of grouping the “wrong” appearance of the circle cannot be considered as described by the subjects only on the basis of the similarity principle, unless the principle of Prägnanz with its ambiguities is invoked. Grouping by similarity/dissimilarity can only create mutual segregation between the two groups as it can be appreciated in the rows and columns of Figures 3B,C.

This kind of result is strongly expected in Figure 4C, where the center of the pattern of Figure 4B is filled by an empty circle. Therefore, the filled circle appears now like an “intruder.” Its dissimilarity and its being beyond the completeness of the pattern, namely beyond its maximal homogeneity (Musatti, 1931) due to similarity, segregates the filled circle from the remainder complete pattern of empty elements.

Segregation is, under these conditions, stronger than the ones of Figures 3B,C, where, although the rows and columns are segregated, they belong anyway to the large squared pattern that includes them anyway. Differently, the filled element of Figure 4C is a single element in the way and its singularity, dissimilarity and loneliness appears to be increased by filling it with a red color (Figure 4D). Whereas the increasing of dissimilarity enhances the possibility of the single circle of being perceived as an intruder; it also highlights *ipso facto* the completeness and homogeneity of the pattern of empty elements. Again, this is the complementation of opposite dynamics (being dissimilar/intruder vs. being homogeneous/complete) previously described.

### Beyond the Gestalt approach

On the basis of the previous phenomenal results, two main problems emerge. The first concern is related to the role of similarity aimed to rule only “what is or stays with what.” It follows that the dissimilar elements of Figure 4B (the filled circle on one side and the empty circles on the other) should appear as belonging to separate groups. In spite of their dissimilarity, however, they appear coupled so that they can influence and define each other. This is clearly the case of the “intruder” of Figure 4C. As a matter of fact, its being “intruder,” i.e., different, depends on the homogeneity of the surrounding empty circles, whose homogeneity is highlighted by the dissimilarity of the filled circle. This suggests that dissimilarities are enhanced and mutually reinforced.

The second concern is related to the role of the center of a group of elements. Although the phenomenal results of Figures 4A,B seem to support the power of the center; on the other hand, they also suggest an alternative view, according to which, the complementation similarity/dissimilarity might describe more appropriately the phenomenal dynamics of the center and its emergence. Whether the center attracts the filled circle and defines its being eccentric or intruder, it is the filled circle that highlights the center and determines its attraction attributes.

Once more, one defines and accentuates the other and *vice versa*. The center and the filled circle are dynamically coupled, so that the meaning of one is defined and reinforced by the meaning of the other. This suggests that the power of the center should be reconsidered. In reality, it is not like an *a priori* force field attracting or retaining a circle, considered as a test body placed within an electromagnetic field, but it is considered as cause and effect at the same time. Additionally, it precedes and follows the formation of the “eccentric” and of the “intruder,” which in their turn precede and follow the formation of the center of mass. This entails that coupling, although related to grouping as studied by Gestalt psychologists, goes beyond it by demonstrating new effects and new possibilities of the visual matter that can create links among elements, generate emerging objects and accentuate hidden properties.

This mutual circular definition could be ascribed to self-organization dynamics of coupling, whose main rules and factors will be described in the next sections.

## Results: from grouping to coupling

### Coupling and accentuation

There are several ways to prove the role and the independence of coupling as a new kind of perceptual organization beyond grouping. It can be done, for example, by demonstrating that coupling and grouping obey to different rules, one can be pitted against the other and both kinds of organization predict different results.

**Figure 5:**
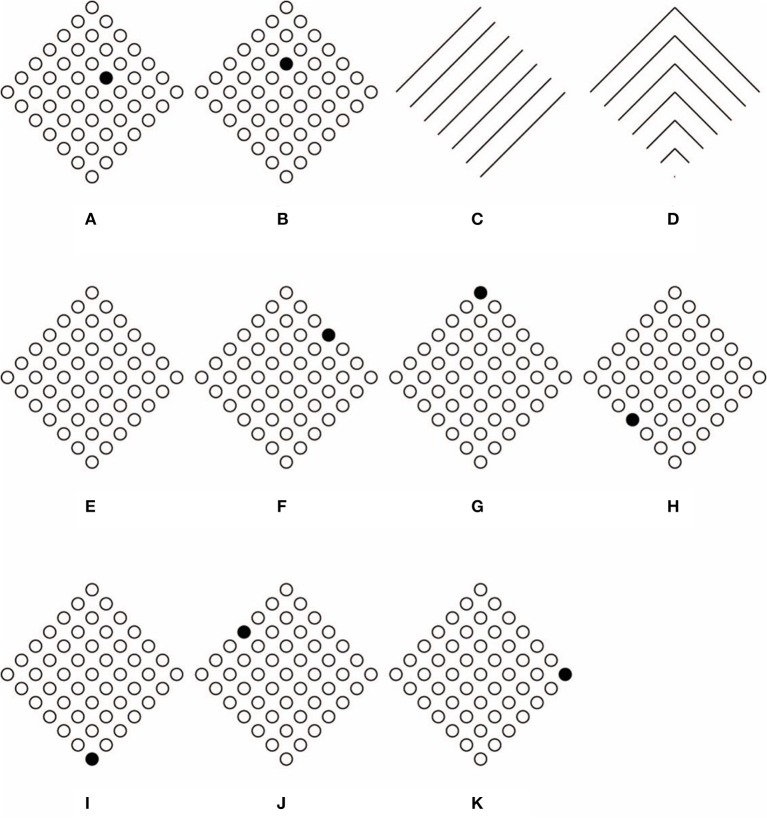
**The dissimilar element defines and punctuates the directions of the grouping of the empty circles**. Conditions **(C,D)** illustrate the directions of the grouping of figures **(A,B)**. Condition **(E)** is a control. The dissimilar element acts like a vector and its location makes figure change their direction **(F–K)**.

By returning to Figures 2C,D, when the geometrical rotated square is made up of circles, the following related variations are useful to understand the different nature of the two kinds of organization. In Figures 5A,B, the shift of the filled circle of Figure 3D of one position within the nodes of the net of circles, respectively toward the upper right side and the upper angle, reveals no attraction toward the center, contrary to what expected from Arnheim's theory. Rather, what emerges, in this particular condition, is the tendency to reorganize the grouping of the whole net of circles according to the position of the filled circle, that in both cases appears to beat, like a musical accent, the directional symmetry of the whole pattern. This suggests the following general principle, all else being equal, the elements tend to group in the same oriented direction of the dissimilar element placed within a whole set of continuous/homogeneous components (Pinna and Sirigu, 2011). In fact, the discontinuous element behaves like an accent or a visual emphasis within a whole. The oriented directions can be schematically represented as illustrated in Figures 5C,D.

In favor of this general statement, it is worth reporting a noticeable tendency observed in 70% our subjects, which was that to rotate the head of 45° in the direction aligned with the axis of symmetry passing through the filled circle of Figure 5A. The rotation of the head is not necessary for Figure 5B, clearly anchored to the location of the filled circle in the horizontal-vertical axis. This effect can be better appreciated by comparing Figures 5A,B with a control (Figure 5E).

By moving further, the filled circle from the center toward the boundaries of the diamonds, the salience of the described effects increases accordingly as shown in Figures 5F,G.

The most important implication of these preliminary results is that the dissimilar element, similarly to an orchestra conductor, defines and punctuates the directions of the grouping of the circles. Indeed, the dissimilar element is perceived like the converging point and the terminal point of the oriented direction induced by it. The starting point of the oriented direction is placed on the circle located at the opposite pole from the accent. The direction, the starting and ending points of the accent likely depend on the directional symmetry induced by it. In short, the dynamics of the dissimilar element can be considered as acting like a vector in an acceptation analogous to that used in physics but existing only in the phenomenological domain. The suggested vectors can be clearly perceived by comparing Figures 5F,G with Figures 5H–K, where the location of the dissimilar element is pole apart or placed in the contiguous side or angles. In this way, vectors can be easily seen as changing direction, and starting and ending points as the location of the dissimilar circle changes. To better appreciate the effect, the figures should be observed separately, not as close as illustrated in Figure 5. This remark will apply to all figures illustrated in this work.

This hypothesis, although apparently similar to the notion of force field suggested by Arnheim, is indeed alternative to it. As a matter of fact, in Arnheim's theory the filled circle is considered as a test body, i.e., totally subjected to the force field generated by the center and radially arranged centripetally or centrifugally. Moreover, the vectorial dynamics here suggested are triggered by the dissimilar circle that defines the organization of the multiplicity of the surrounding made up of homogeneous elements.

**Figure 6:**
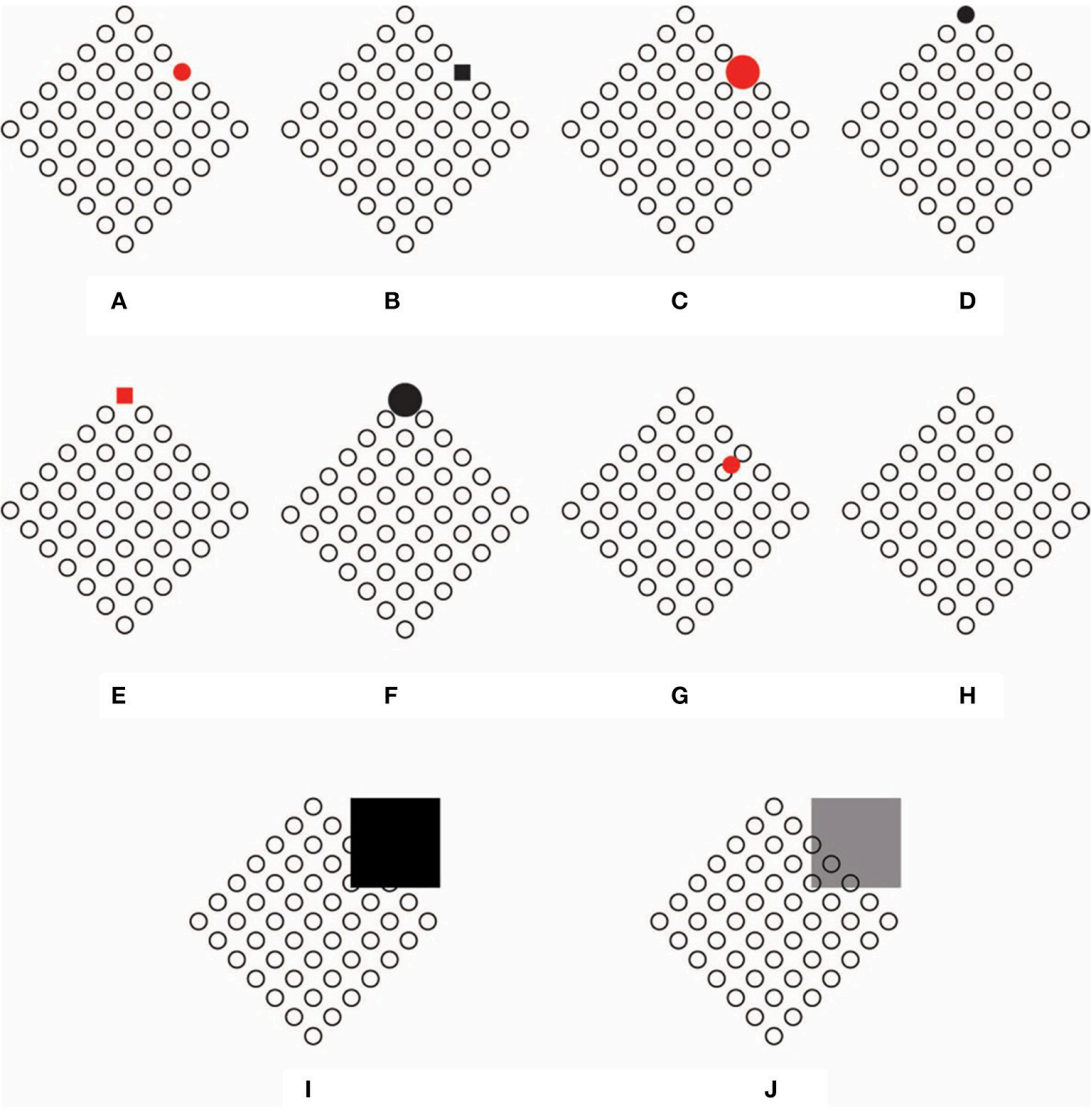
**Grouping organization changes its strength as the salience of the dissimilar element changes**. The location of the dissimilar accent affects the grouping of element in the direction of the upper right side **(A–C)**. The location of the dissimilar accent affects the grouping of element in the direction of the upper angle **(D–F)**. The dissimilar accent is introduced within the group of empty circles **(G)**, as a gap **(H)**, as an amodal completion of part of the whole array of circles **(I)**, as an overlapping transparent layer **(J)**.

If the dissimilar element is the crucial element, it follows that the resulting grouping organization is expected to change its strength as the strength of the dissimilarity of the element changes. The dissimilarity of the target component was varied in shape (circle-square), color (black-red), and size (small–large; cf. Figures 6A–C for some combinations) for a total of 8 stimuli × 2 conditions (diamond and rotated square). The experimental tasks were preceded by a few minutes training when the observers familiarized with the Gestalt grouping principle of similarity as illustrated in Wertheimer (1923) and in Kanizsa (1980). The result of the phenomenological and scaling task showed that all these variables affected the strength of grouping organization as follows. First of all, the location of the dissimilar element influenced the grouping of the empty circles as expected, namely in the direction of the upper right side or of the upper angle. Moreover, by increasing the salience of the dissimilar element, the strength of grouping in the two main directions (upper right side or upper angle) changes accordingly.

Despite the aim of this work is not to measure psychophysically in details the role and the different kinds of dissimilarity of the critical element in influencing the perceived grouping, more quantitative data emerged from the magnitude estimation task. The eight stimuli for each condition (dissimilar element placed on the upper right side or upper angle) were shown randomly all at the same time to two different groups of 14 subjects. Each condition was judged in a separate experiment given the clear results of the phenomenological and scaling tasks. The task was to scale the relative strength in percent of the grouping in the two main directions, where 100 is the maximal strength perceived and 0 the minimal, i.e., the control condition without dissimilar element as shown in Figure 5E. This procedure was repeated in the next conditions.

The results of the magnitude estimation corroborated the phenomenological and scaling ones. More particularly, 2 three-way within-subjects ANOVAs (variations of shape × color × size) revealed significant variations of the strength of the grouping occurring in both the upper right side and in the upper angle directions due to the three variables: shape [*F*_(1, 13)_ = 8.3, *P* < 0.05 for the upper right side condition, *F*_(1, 13)_ = 7.5, *P* < 0.05 for the upper angle side condition], color [*F*_(1, 13)_ = 7.6, *P* < 0.05 for the upper right side condition, *F*_(1, 13)_ = 8.4, *P* < 0.05 for the upper angle side condition], and size [*F*_(1, 13)_ = 7.9, *P* < 0.05 for the upper right side condition, *F*_(1, 13)_ = 6.8, *P* < 0.05 for the upper angle side condition]. All the interactions between the three factors were also significant (*P* < 0.05).

The outcomes of the three tasks suggest that the dissimilar element does not behave like a test body but it assumes a more significant and active value within the dynamics of the perceptual organization becoming a sort of leading actor.

The salience of the dissimilar element can also be handled as shown in Figures 6G–J, where dissimilarity is introduced as a circle in the way within the group of empty circles, as a gap or as an absence in the continuity of the nodes, as an amodal completion of part of the whole array of circles (see Pinna, 2013) or as an overlapping transparent layer [*F*_(3, 39)_ = 6.4, *P* < 0.05].

The oriented directions of the resulting grouping is only one of the two main effects imparted by the accentuation due to the dissimilar component. The other phenomenal outcome is related to the perceived global shape of the pattern, that is again a consequence of the accentuation of the directional organization of the elements and of the whole object. Since accentuation is focused on sides and angles, the related phenomenal properties are “sidedness” and “pointedness.” The accentuation of the sidedness goes together with the perception of a whole square rotated by 45°, while the accentuation of the pointedness goes with the perception of the diamond, as reported in Section Introduction. The results related to the strength of the “rotated square” and of the “diamond,” under the same conditions as previously described (Figure 6), were not significantly different from those related to grouping. This suggests that grouping and shape perception, under our conditions, emerge jointly and that the one is mutually anchored to the other.

These results demonstrate that the power of dissimilarity can be much stronger than that of similarity. Moreover, while the former induces homogeneity, the latter elicits changes and discontinuities that spread filling the surrounding homogeneous field of components. This implies that, whether similarity is one of the main principles of grouping, defining “who is with who and who is against who,” namely grouping and segregation among groups, on the other hand, dissimilarity is the source of a new kind of organization that couples and connects similarities and dissimilarities in a new way and at a different perceptual level. In relation to this, we suggest that coupling allows the binding of many dissimilar kinds of elements, which would otherwise take place in isolation if considered only on the basis of grouping. In the next section, this thesis will be further explored in the light of this new phenomena.

### Similarity vs. dissimilarity and proximity vs. remoteness

Previously, the metaphor of the orchestra conductor was introduced to better show the incipit and the start elicited by the dissimilar component. This metaphor is effective also in the geometrical acceptation. In fact, just as the conductor operates from a location separated from the orchestra, the dissimilar component, which punctuates and structures the set of elements, possibly does it even better from a separate location. It is perhaps no coincidence that the orchestra conductor, which is the dissimilar component of the orchestra, is placed closer but separated from the orchestral members.

**Figure 7:**
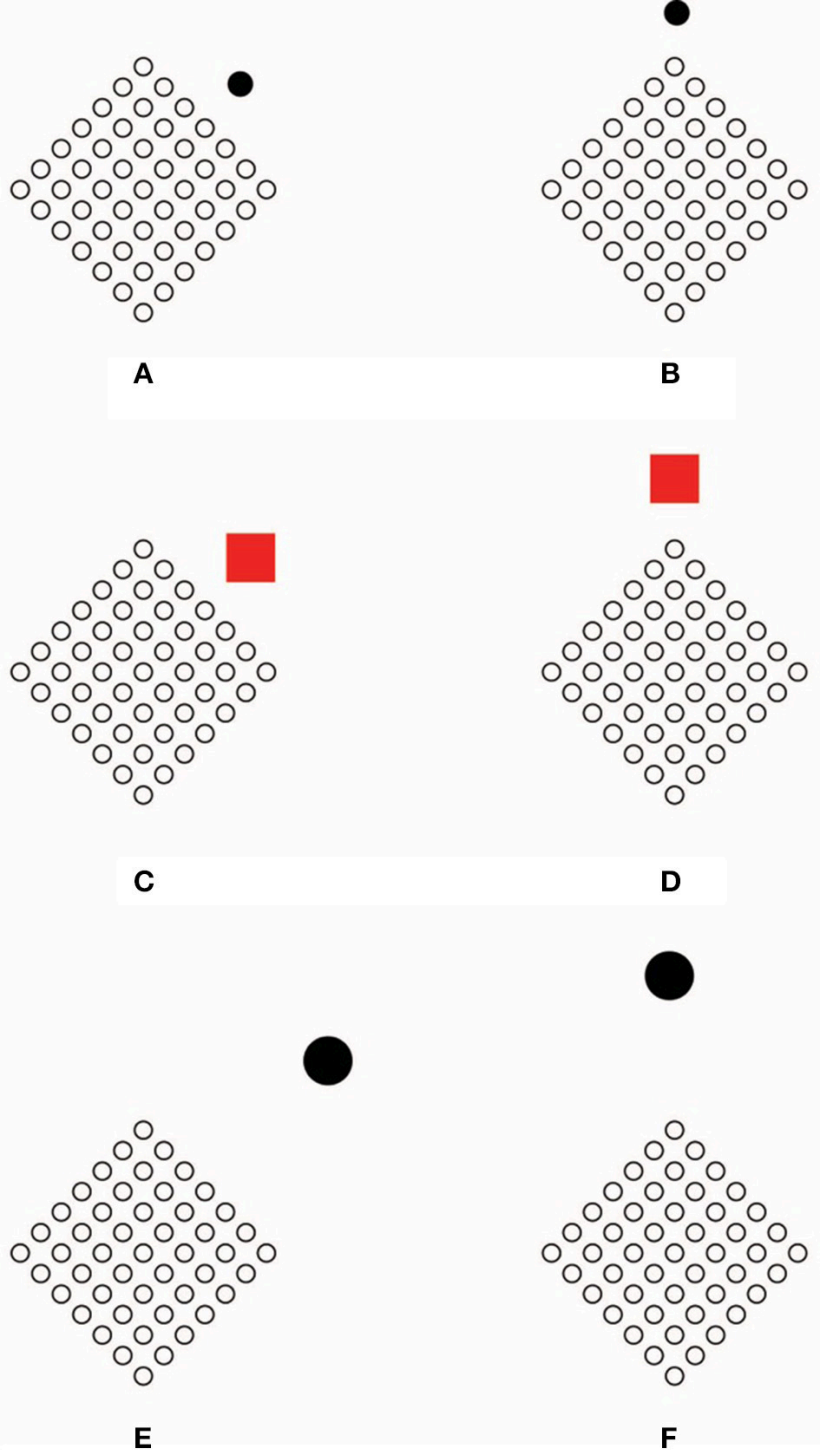
**The increasing of the distance between the two components of coupling does not weaken significantly the resulting effects: (cf. A,B with C,D with E,F) grouping and accentuation of the sidedness/rotated square (A,C,E) or pointedness/diamond (B,D,F)**.

In Figures 7A,B, the spatial separation (in relation to the on-boundary) between the group of empty small circles and the filled one does not prevent coupling between them as well as it does not block the reorganization of grouping [*F*_(1, 13)_ = 8.8, *P* < 0.05, the difference with on-boundary condition was not significant] and the accentuation of the sidedness/rotated square (Figure 7A) or pointedness/diamond (Figure 7B) in the direction conducted by the large circle. An even stronger effect is perceived in Figures 7C,D, where chromatic and shape variations enhance these effects as demonstrated in the previous section [*F*_(1, 13)_ = 7.3, *P* < 0.05].

The increasing of the distance between the two groups does not weaken significantly the resulting effects as shown in Figures 7E,F. Under these conditions, the grouping principle of proximity is also involved, although it is expected to increase the phenomenal ungrouping of the two main components of each pattern. Therefore, by increasing ungrouping which is, first of all, due to the similarity of one group of empty circle and their dissimilarity with the filled element, and, secondly, to their proximity against the larger distance of the segregated element, the expected result should be a complete absence of connections among the two groups. On the contrary, they are clearly coupled and influencing each other. The main thesis here suggested is that the strength of coupling increases *a fortiori* as the ungrouping increases. As a matter of fact, coupling puts together dissimilar elements that cannot be grouped and that *ipso facto* remain ungrouped.

By demonstrating that the binding between dissimilarities occurs at long distances, these results corroborate the notion of coupling as different from grouping. Undoubtedly, while grouping is ruled by similarity and proximity, coupling appears to be more effective under dissimilarity and remoteness. These opposite rules emphasize the fact that they belong to different kinds of perceptual organization.

### Subject or object of the accentuation? who's who?

A more careful phenomenal evaluation of Figures 7C,D reveals that, even though the red squares influence grouping and the holistic shapes of the empty circles, they are conversely influenced by the empty circles. By comparing the shape of the two squares (Figures 7C,D) the one on the right manifests sidedness more strongly, i.e., it appears like a square, while the other on the left shows pointedness and appears like a diamond. This means that one element, either the square or the array of empty circles, accentuates the adjacent component (side or angle) of the other. This can be generalized by saying that within coupling the accentuation effect between the two components is reciprocal.

This result is in some way expected if we think to the reference frame effect that is usually considered to be imparted by the larger component. The well-known Kopfermann's effect clearly supports the dependence of an object shape on the frame of reference (Kopfermann, 1930; see also Gibson, 1937; Witkin and Asch, 1948; Antonucci et al., 1995). However, this is in contradiction with all the conditions previously shown, where the accent, i.e., the subject of accentuation, was not the frame of reference.

**Figure 8:**
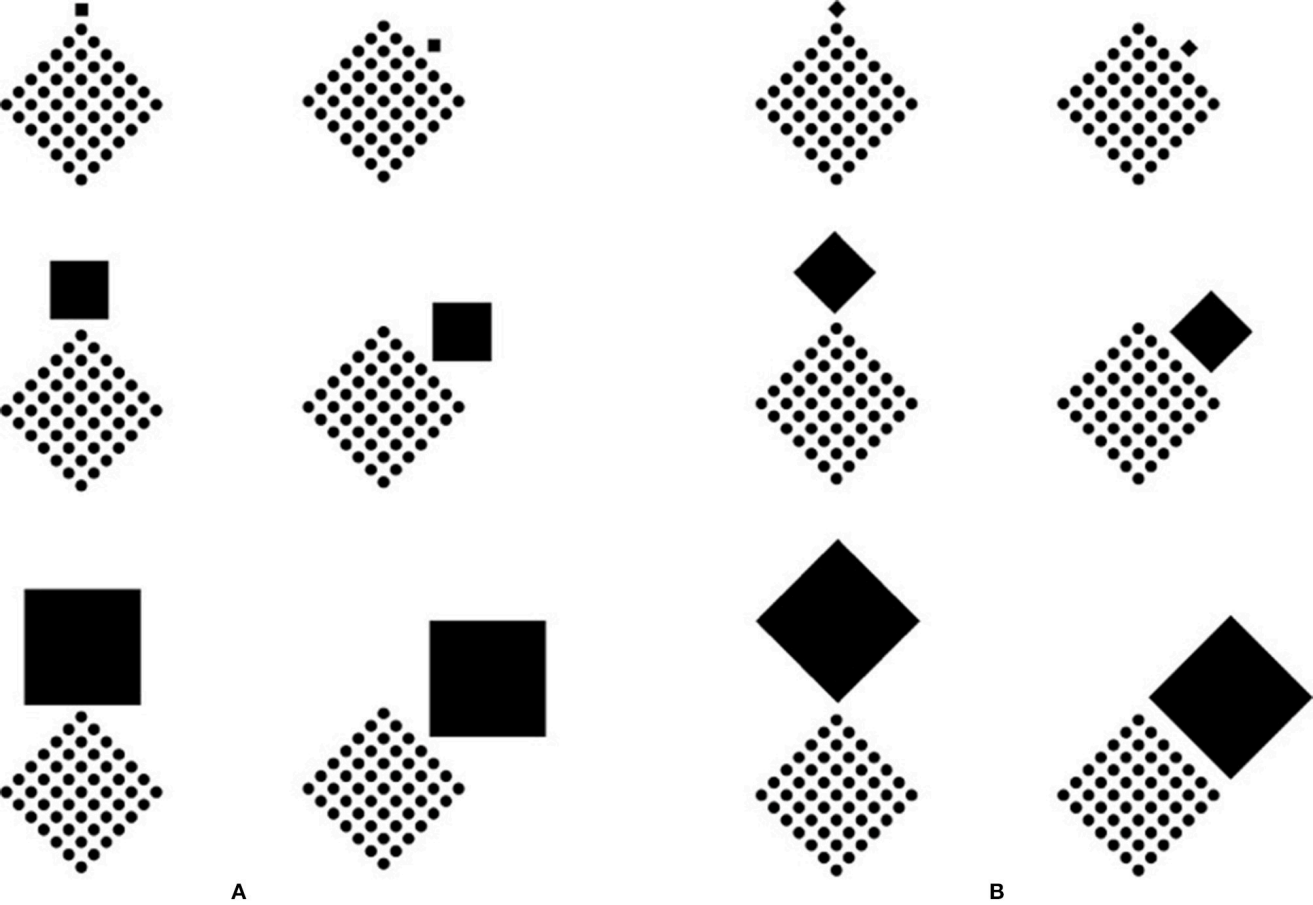
**By comparing all the conditions, both the dissimilar element [a square in (A) and a diamond in (B)], and the set of circles influence each other in the perceived grouping (for the circles, see the text) and in the shape [for both the array of circles and the dissimilar element, e.g., the large black shape is perceived as a square in the first column of (A) and in the second of (B), and as a diamond in the second column of (A) and in the first of (B)]**.

In the light of the specific shape effect described for the squares of Figures 7C,D, the questions are: can this peculiar result be generalized from all the others here considered (see also Grossberg and Pinna, 2012; Pinna, 2012a,b)? More generally, who is the subject of accentuation and who is the object accentuated? In terms of reference frame, the answer is implicit and it is referred to the larger and including component. Within the notion of coupling the problem of the inner dynamics is more complex because it contains two dissimilar counterparts to be coupled. The term “coupling” subsumes the idea of a reciprocal interaction and influence in between two dissimilar elements. They are two and one at the same time: two, since they are not perceived as grouped in rows and columns as in Figures 3B,C; one, since they strongly influence each other as our result demonstrated. If this is true, the problem of the reference frame in terms of size within the couple, i.e., the fact that one is larger and including the other, is misleading. Figures 8A,B show some examples of the described mutual effects occurring by changing the size of the square. Two conditions are depicted: in the first one (Figure 8A) the dissimilar element is the square; in the second one (Figure 8B), the dissimilar component is the diamond. Both could be perceived as squares or diamonds in relation to their location near the array of small circles. These two conditions are divided into two sub-conditions related to the position of the dissimilar element. Finally, they are all varied in three sub-parts where the dissimilar component has three different sizes: small, medium, and large.

All variables involved manifest a clear effect both on grouping and on shape. There is an effect due to the positions of the dissimilar element, which has been already discussed in the previous sections. In details, there is an effect of size, also demonstrated in the previous section. The new effect is related to the perceived shape of the dissimilar component in its three different sizes. By comparing all the conditions and, specifically, the perceived shape in terms of square vs. diamond, both the dissimilar element and the set of circles influence each other in the perceived grouping (for the circles) and in the shape (for both the array of circles and the dissimilar element). The dissimilar element rules the direction of the grouping of the circles, according to our previous descriptions, and the shape of the array of circles, by highlighting sidedness or pointedness. Mutually, the array of circles defines the perceived shape of the dissimilar component in terms of rotated square or diamond on the basis of their reciprocal location. Therefore, when the array highlights the sidedness of the dissimilar element, it appears as a rotated square, while, on the contrary, when its pointedness is accentuated, it appears as a diamond.

This implies that the answer to the starting question (who's who?) is to be found in both components of the couple.

The unidirectional organization of grouping and of the force field suggested by Arnheim cannot account for the most complex interaction of coupling, whose aim is to create connections (couplings) among different objects within the visual world. This represents a powerful enhancement of possible combinations, which go much beyond those suggested by figure-ground segregation and grouping. In the next sections some biological implications of coupling and its effect in evolutionary terms will also be discussed.

The results of this and of the previous section can be accounted for by another alternative hypothesis, according to which the role of the accent is to focus the visual attention on a short range region surrounding the dissimilar element. The restriction of the attention highlights visual attributes that are potentially perceptible and it also shows groupings related to all the elements involved within the zoomed short region.

Without denying the role of visual attention in these kind of effects (see Grossberg, 1997; Grossberg et al., 2001; Grossberg and Pinna, 2012), the mutual effects shown in Figure 8 suggest that the change in the directional organization of elements and the highlight of the sidedness and the pointedness are mostly related to a more general problem of perceptual organization considered in terms of coupling. The spontaneous rotation of the head according to the location of the accent, together with the two-way influence, weaken the role of attention. Moreover, if attention is the basic process involved in our conditions than it should also be the basic process in the dynamics of grouping studied by Gestalt psychologist. In fact, when similarity puts together elements in rows or in columns as shown in Figures 3B,C, they attract and polarize the focus of attention in the direction of grouping. A similar argument can be applied to our conditions.

The crucial point is that attention is surely involved. However, not only is it involved to organize but also it is essential to focus on the results of the organization that precedes its involvement. In other words, the focus of attention is attracted by the dissimilar element and consequently by the results of accentuation, highlighting them even further. This counterargument can be tested in the conditions illustrated in the next sections.

A final remark is related on how easy and spontaneous it is to connect and relate the visual attention to the accent, to its meaning and to the fact that it belongs to the dissimilar element, which is an attractive visual element *per-se*. This could be indeed the source of fusion and confusion between the notion of accent and attention.

### Different groupings due to different accents: sliding motion in depth

**Figure 9:**
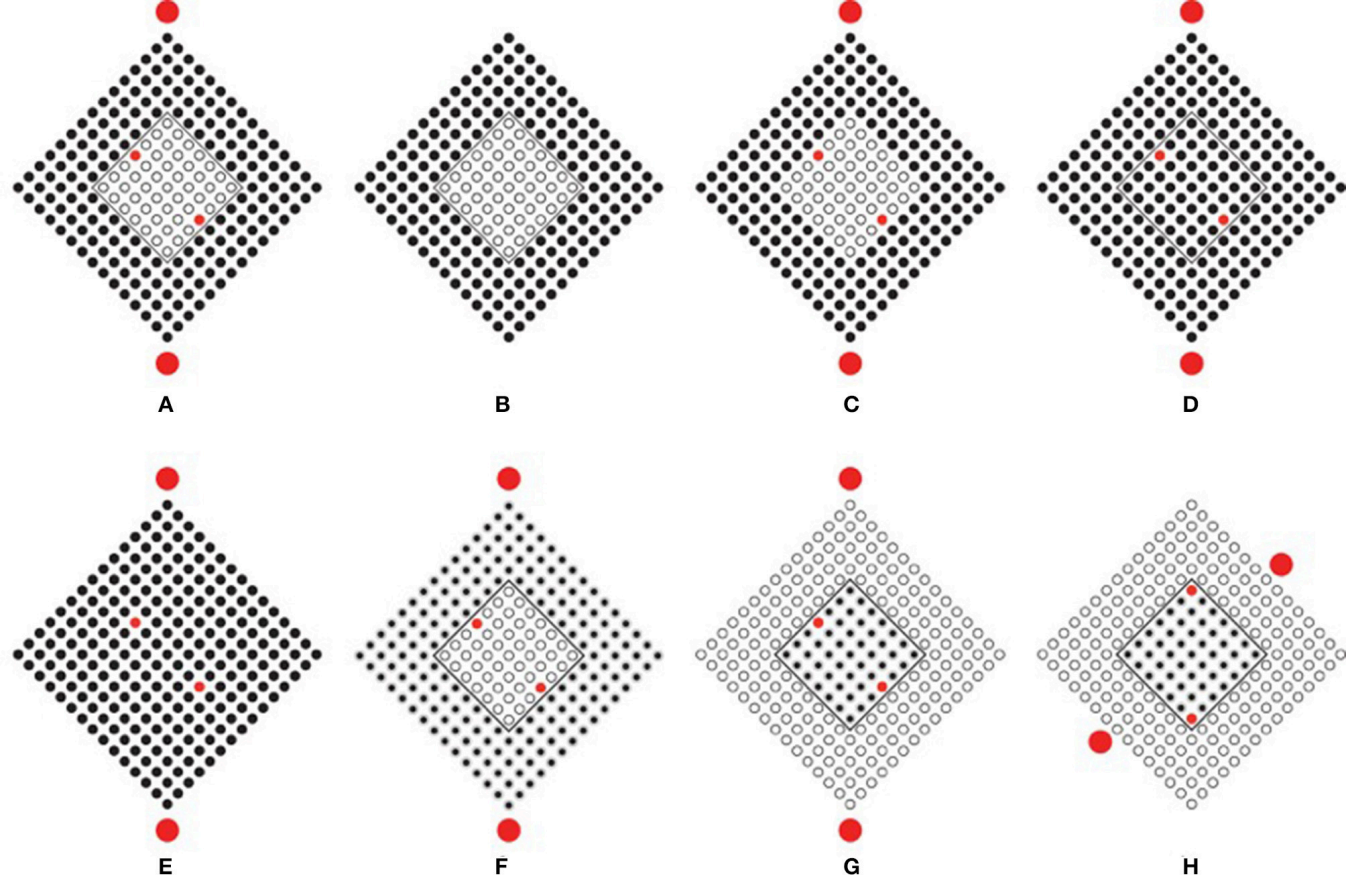
**In spite of the belongingness of all circles to the same large array (see B as a control), the presence of accentuation along different directions reorganizes the two nested arrays according to the accent (vertical for the large array and oblique for the inset one in A,C,D,E,F,G, or oblique for the large array and vertical for the inset one in H)**.

The necessary distinction between grouping and coupling is corroborated by the conditions illustrated in Figure 9A, where two arrays made up of empty (the inset one) and filled circles are, respectively accentuated in different directions by red circles along two antipodean sides and angles. In spite of the belongingness of all circles to the same large array, the presence of their accentuation along different directions reorganizes the two nested arrays according to the accent. The control without the effect of reorganization of grouping is illustrated in Figure 9B. It can be argued that the large inner frame, playing a basic role in separating the two arrays on the basis of the principle of common region (Palmer, 1992), might be responsible for the main effect. In Figure 9C, despite the absence of the frame, the effect is still perceptible even if slightly weaker. It cannot be denied that the frame plays a role in the separation of the two frames although this role is not crucial. If the frame is reintroduced but the inner empty circles are filled, as shown in Figure 9D, the reorganization of grouping due to the accents is further weakened even though it is still present. It is also still perceptible in Figure 9E, when the frame of Figure 9D is absent. In fact, in the region around the inner accents the organization follows their direction, while in the surrounding region grouping organization follows the external large red circles. These phenomenological results are confirmed by the ones of the magnitude estimation task [*F*_(4, 13)_ = 5.3, *P* < 0.05].

The strength of this result can also be enhanced as illustrated in Figures 9F–H. Most of these conditions reveal the illusion of sliding motion in depth (Pinna, 1990, 2009; Pinna and Spillmann, 2002a,b; (Pinna and Spillmann, 2005)), perceived now stronger than in the original one (without red circles, see Pinna and Spillmann, 2002b), due to the accents that orient in different directions the arrays of elements [*F*_(4, 13)_ = 5.7, *P* < 0.05].

Under these conditions, coupling and accentuation can be considered as the main responsible factors for these results.

### Similarity against accentuation and grouping against coupling

To demonstrate the role of coupling beyond grouping, it is crucial to demonstrate that the former can be pitted against the latter and that both kinds of visual organization predict different results.

**Figure 10:**
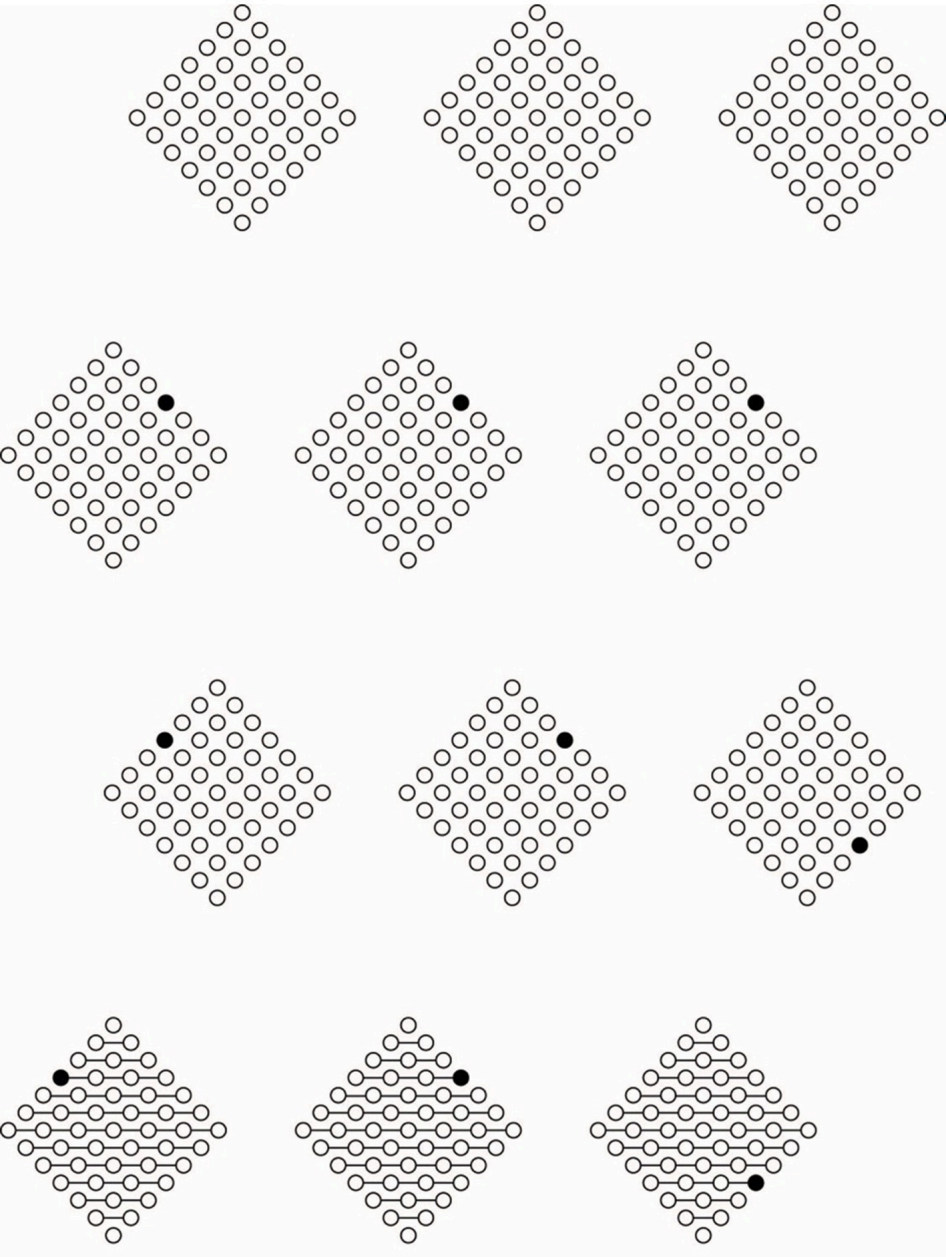
**Configural orientation effect (first row)**. Accentuation is pitted against configural orientation effect (second row). The patterns appear rotated in different directions according to the location of each accent (third row). Same outcome as (third row) when accentuation is pitted against the element connectedness principle (fourth row).

A first case where the two processes of perceptual organization can play against one another is pictured in Figure 10. In the first row of Figure 10, the configural orientation effect (Attneave, 1968; Palmer, 1980, 1983, 1985, 1989, 1999; Palmer and Bucher, 1981) demonstrates that the perception of the local spatial orientation is determined by the global spatial orientational structure, which can be considered as a special case of the Gestalt grouping principle of good continuation. Since the global orientational structure follows the angles of each pattern of small circles, the expected results of the perceived shape is “diamonds” rather than “rotated squares.” This result is clearly perceived. In the second row of Figure 10, the same global spatial orientational structure is now segmented and reoriented by the filled circles in each pattern. This means that accentuation is pitted against the configural orientation effect. Between the two principles, coupling and its resulting accentuation stand over the other. What actually emerges are in fact “rotated squares” in each pattern. The first row of Figure 10 can now be used as a control. In the third row of Figure 10, the square patterns appear rotated in different directions according to the location of each accent within the pattern, thus breaking the global spatial orientational structure and good continuation. The same outcome (the fourth row of Figure 10) persists, although weaker, also when a further principle of grouping (element connectedness, Palmer and Rock, 1994) is synergistically added to the global spatial orientational structure and both are pitted against the accentuation.

**Figure 11:**
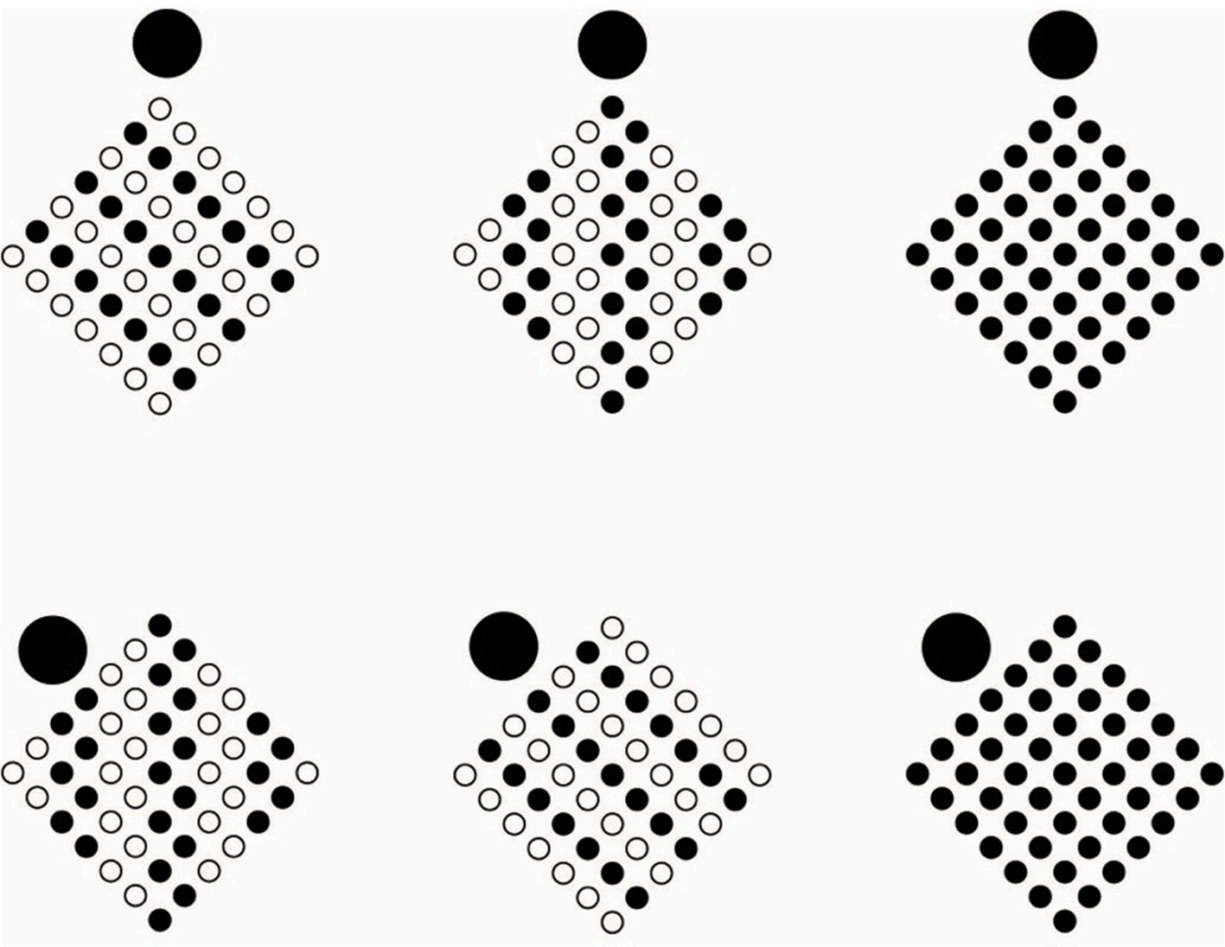
**Similarity principle pitted against Accentuation principle**.

Other conditions demonstrating a stronger conflict between grouping and coupling are pictured in Figure 11. The principle of similarity is now pitted against the principle of accentuation. Before describing the results, it is worthwhile to note that the principle of similarity is to the principle of accentuation as grouping is to coupling. In the two-first columns of Figure 11, the accent plays respectively against and in favor of the principle of similarity. In the third column a control is illustrated. The outcomes of the first column demonstrate that the two principles and organizations undermine each other, therefore the perception of a “diamond” rather than a “rotated square” remains in balance if it is compared with the controls. As regards to the second column, the two principles operate synergistically, thus the resulting effect is enhanced. These phenomenological outcomes are corroborated by the ones of the magnitude estimation [*F*_(2, 13)_ = 4.4, *P* < 0.05 and *F*_(2, 13)_ = 4.8, *P* < 0.05].

These results are points in favor of the counterargument against the role of visual attention. In fact, the results of the first column cannot be interpreted in any way as elicited by attention only. The attention involved under these conditions is of the same amount and strength of the attention involved in pure grouping conditions. Again, if attention is crucial in coupling it should also be crucial in grouping.

### Similarity as accentuation

Although the possible instances of accents and their attributes are not the main purpose of this work, some answers to the question “what can be an accent?” can be provided. Previous studies already explored the nuanced large set of accents (Pinna, 2010b, 2012a), but in the context of this work, it is necessary to mention the fact that even though similarity among elements is a grouping principle, it can also be considered as an accent. This distinction can be very useful to provide more details concerning the differences between grouping and coupling.

If similarity is considered as a grouping principle, it defines what stays with what by putting together elements with maximal homogeneity and nothing else. If, instead, similarity is intended as an accent, then its directional organization behaves similarly to the filled circle of the previous sections, which, by inducing a directional accentuation, imparts to different elements grouping and shape accentuations. The only difference lies in the fact that similarity is in this case like dissimilarity is in coupling. Moreover, while the dissimilar filled element couples with the set of empty circles, on the other hand, the result of similarity couples with each single element and with the whole pattern of components. The same logic can be applied to other grouping principles, such as proximity and good continuation, since they involve directional organization.

**Figure 12:**
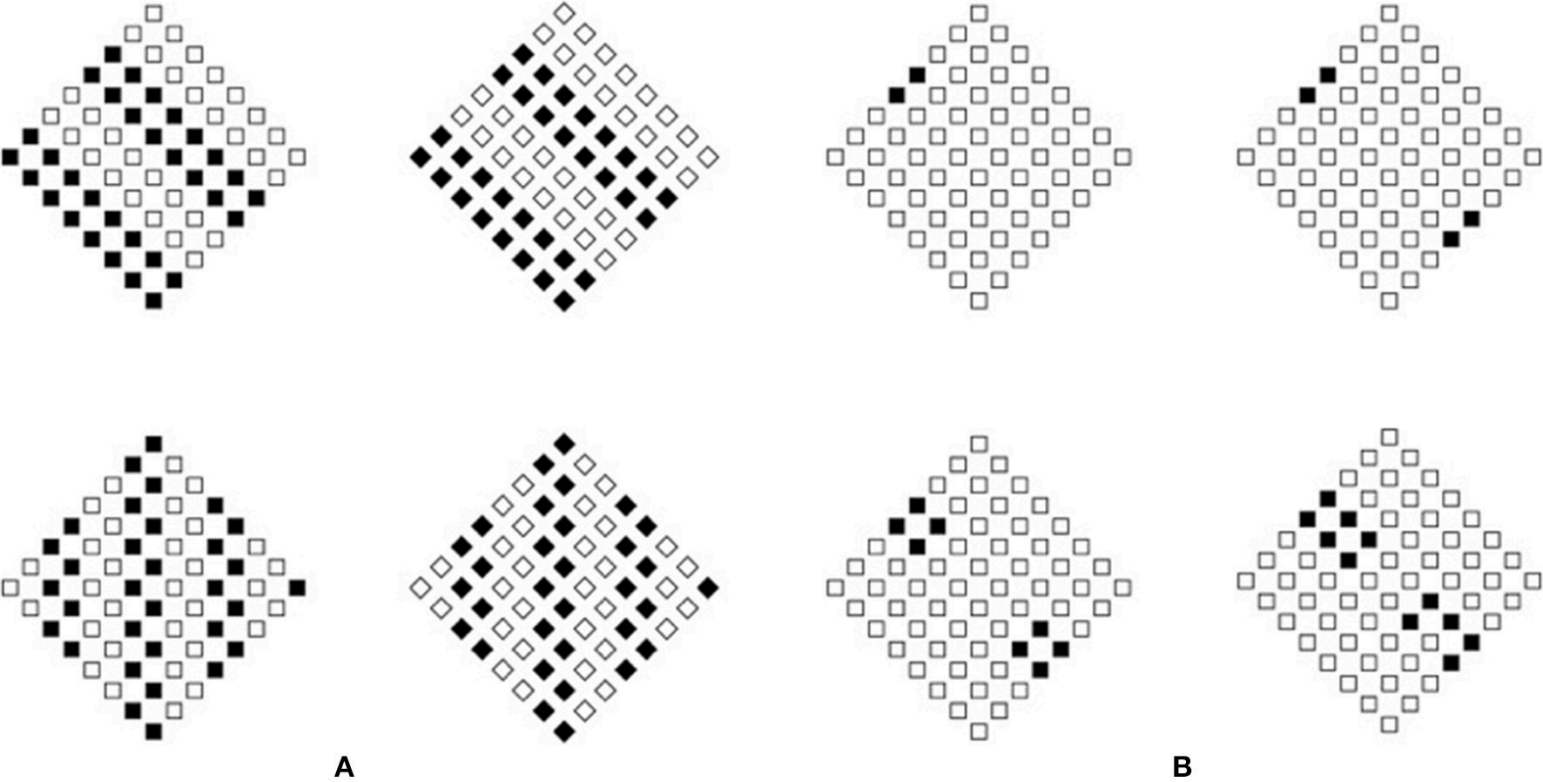
**The principle of similarity considered both as a grouping and as a coupling principle (A)**. A gradual transition going from the dissimilar accent to the principle of similarity considered as a coupling principle **(B)**.

Figure 12A clarifies this distinction by showing four conditions ruled by the principle of similarity seen either as a grouping principle or as an accent. When it is considered as a grouping principle, its outcomes are oblique stripes, in the first row, and vertical stripes, in the second, nothing else. However, if similarity is seen as an accent, then its result can influence both the shape of the single elements and the shape of the whole object. In the first row, the elements of the pattern on the left are small squares but, on the basis of the principle of similarity, they are accentuated in the oblique direction, i.e., in the direction of the angles and thus into the pointedness. Therefore, the expected result is “diamond.” In short, squares appear like diamonds. However, if we consider the effect of similarity on the whole patters, the result is a “rotated square.”

The right pattern of the first row is made up of small diamonds, but, due to the same logic, they are perceived like rotated squares similarly to the whole pattern. In the second row, by applying the same logic, the pattern on the left is made up of squares that appear like squares, while the whole pattern is perceived like a diamond. The pattern on the right, made up of diamond, shows diamonds both in the elements and in the whole object.

In summary, the four phenomenal combinations are: (i) small squares that appear like diamonds and a whole diamond perceived as a rotated square; (ii) small diamonds seen as rotated squares within a large rotated square; (iii) small squares appearing as squares within a diamond seen as such; (iv) small diamonds seen as diamonds within a diamond perceived as a diamond.

The simplicity and salience of these results cannot be accounted for only in terms of similarity as a grouping principle without creating confusion with the meaning of grouping. For this reason, we propose the necessity to introduce the notion of accentuation as a coupling principle.

The strong theoretical connection between dissimilarity accent principle and similarity coupling can be demonstrated through a gradual transition, which, going from the dissimilar accent as considered in the previous section, reaches similarity considered in terms of coupling. In Figure 12B, four conditions using the same basic stimulus (first row left) of Figure 12A are gradually completed with filled squares. The first condition can be considered as the dissimilar element studied in the previous sections, while the fourth condition is analogous the one ruled by the principle of similarity. These results demonstrate no differences in the perceived shapes that are of the first kind.

### Accentuation vs. salience and coupling vs. medium-range grouping

In the previous sections “accentuation” was used in an acceptation different from the term “salience.” Even though the differences between the two terms have been already discussed in the Introduction Section it is necessary to trace a more definite separation between them in the light of the new figures previously described. In this section, also the difference between the visual dynamics of coupling and the medium-range grouping will also be discussed.

As previously described, visual salience is the distinct subjective perceptual quality that makes some elements or attributes pop out from their neighbors in such a way that they immediately grab our attention. Visual salience is also involved in figure-ground segregation, making the figure emerge from the surrounding background. This entails that visual attention is attracted to salient stimuli, that can be considered important for complex biological systems necessary to rapidly detect any potential prey, predators, or mates within the molteplicity of articulations of the visual world (Itti, 2007). However, given the high computational complexity of the salient stimuli, the main solution adopted in nature is to restrict the complex object recognition process to a small area or to a few objects at a time. The many objects or areas in the visual scene can then be processed by serializating the analysis of the visual scene. This is made by means of mechanisms of visual attention that behave similarly to a spotlight, shifting to, and highlighting different sub-regions of the visual world, so that one region at a time can be subjected to more detailed visual analysis (Treisman and Gelade, 1980; Crick, 1984; Weichselgartner and Sperling, 1987).

In short, due to the perceptual quality of salience, based on perceptual differences, dissimilarities and changes, one element within a large set of elements pops-out and effortlessly and immediately attracts attention (Treisman and Gelade, 1980; Wolfe, 1998).

From these remarks, visual salience is indeed considered as the main attribute of the small circles and squares used as accents in the previous sections. The question is now: how does accentuation differ from visual salience? The main differences that require the introduction of the term accentuation are the following. First of all, saliency is a visual attribute attracting attention and popping out its holder, i.e., the element that appears salient. Accentuation is, instead, a perceptual process triggered and imparted by one or more elements (being salient or not) and highlighting at least one visual attribute of another object that, as such, is referred, linked and coupled with the accent.

Moreover, while the dynamics of salience is focused only or mostly on its holder, the dynamics of accentuation goes beyond the accent itself and is directed and oriented to one or more objects both locally and globally (see the single and global square shapes of Figure 12).

Again, while salience is strongly related with visual attention, we showed in the previous sections that accentuation does not necessarily require attention.

Whereas salience is a visual attribute belonging to one element or component against others, accentuation is a mutual effect going from one to another element and *vice versa* (see Figures 7, 8). In other terms, both components behave like accents and accentuated targets at the same time. They can be sources and targets, causes, and effects.

While salience is an attribute inducing only a popping out effect of the object being salient, accentuation can induce a larger variety of effects: perceptual grouping, pointedness or sidedness, figure-ground segregation (Pinna and Sirigu, 2016), dynamic and motion effects (Pinna and Sirigu, 2016), visual rithms (Pinna and Sirigu, 2016), gravity and countergravity effects (Pinna and Sirigu, 2016), organic organization (Pinna, 2012a,b, 2015; Pinna and Sirigu, 2016), directional organizations (Pinna, 2015), illusions of musical upbeat suspension (Pinna and Sirigu, 2011) and musical downbeat (Pinna and Sirigu, 2016).

**Figure 13:**
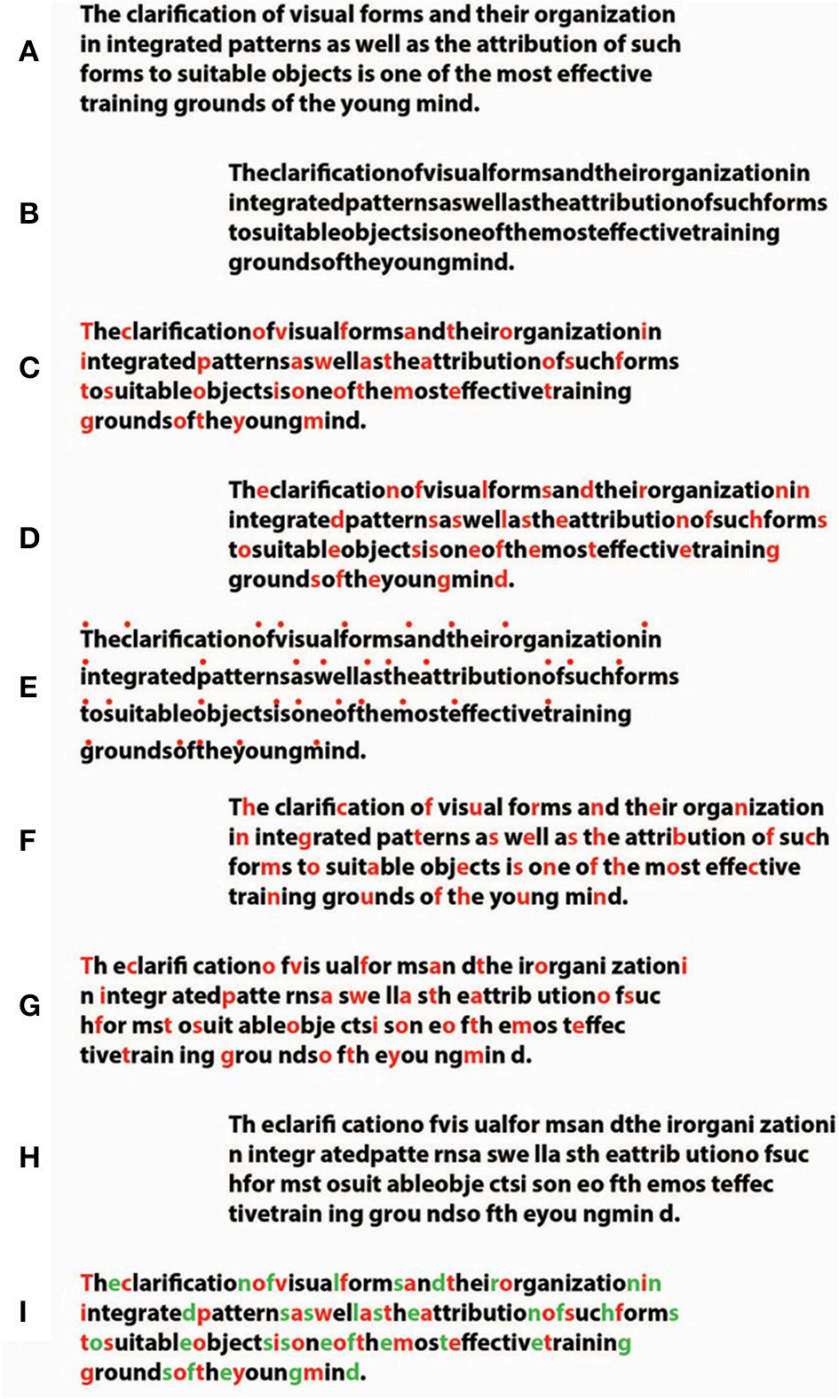
**Accentuation plays a clear role in making it easier (C,D,E,F,G,I, cf. with the controls A,B,H) to read Arnheim's quote**. It also makes the reading faster or induces a delay and improves or makes it worse the comprehension of the text. These phenomenological results are confirmed by the ones of the magnitude estimation task [*F*_(8, 13)_ = 8.2, *P* < 0.01].

In our written and spoken language the term “accent” is an independent sign and object (see Figure 13). Accents are something, not just attributes, but something phenomenally autonomous. Accents are present not only in vision but also in music and both manifest the same independence and autonomy. In other words, the phenomenal status of the accent and of its action, i.e., to accentuate, is phenomenally much more prominent than the salience, which is instead restricted to the domain of perceptual attributes only.

While the dynamics of the accent and the accent itself are comparable to vectors in the acceptation analogous to that used in physics but operating within the phenomenological domain (see Section Coupling and Accentuation), salience can in no way be reduced to a vector.

A further important difference between salience and accent is also the following: while a salient element is not necessarily an accent, i.e., it is not necessarily able to accentuate, the accent is not necessarily a salient element. This is, for instance, the case of the large black squares of Figure 8 that are accents for the dotted global squares and, at the same time, are accentuated by them. None of these components show a clear salience attribute, they do not show differences in salience, although they clearly induce an accentuation effect one another.

All of these specific and unique properties of the accent and of the accentuation justify and made necessary the introduction of these terms (already phenomenologically present in our language) within the context of perceptual organization. As a matter of fact, being the accent like a vector inducing different kind of perceptual organizations among different elements and being accentuation clearly different from the concept of grouping, its role is to put together things that cannot be grouped. The accentuation induces mutual interactions among ungrouped elements that, as a consequence, are coupled throughout their being accent and accentuated.

On the base of these main differences and on the distinctive properties of the accentuation inducing coupling, the answer to the question “How does coupling differ from medium-range perceptual grouping?” can it be easily found? If accentuation induces mutual interactions among ungrouped elements and if coupling emerges through accentuation, then coupling is not a grouping process. This implies that medium-range perceptual grouping and coupling are different processes. As a matter of fact, coupling and grouping are based on principles that are opposite, namely, founded on differences and dissimilarities, the former, and maximal homogeneity, the latter.

### Accentuations in reading

In the previous sections we have shown and analyzed the independence of the accentuation principles from other grouping principles. We have also demonstrated its basic role within coupling organization through its salience in simple geometrical conditions. The question is now: is this principle equally effective in more complex conditions?

The case considered in this section is the reading task. In Figure 13A, an Arnheim's famous quote is reported. The text has been segmented by means of the chromatic accentuation, i.e., by using the similarity/dissimilarity principle in coupling acceptation. The quote has been manipulated by canceling the blank spaces in between words (Figure 13B) and, then, it has been highlighted by different chromatic conditions: Figure 13C by putting in red the first letter of each word; Figure 13D, the last letter; Figure 13E by the addition of a small red circle above the first letter of each word. Moreover, the accentuation has been pitted against the principles of proximity and past experience at the same time, i.e., by breaking each word, separated by a blank space, through accentuation, thus making it difficult to be read (Figure 13F), or, on the contrary, in order to make it readable again, with words broken in the middle by a blank space (Figures 13G,H for a control) and finally, by accentuating each word with two different colors, one for the first, and one for the last letter (Figure 13I).

The results demonstrated that accentuation plays a basic role in making it easier or difficult to read the quote. This can be considered like a masking and an unmasking, disrupting and highlighting effect. The salience of the accentuation can be immediately experienced and it is time consuming. It makes the reading faster or induces a delay. Moreover, accentuation also involves the comprehension of the text by improving it or by making it worse. Related to these results are interesting studies on crowding (cf. Pelli et al., 2007; Pelli and Tillman, 2008; Whitney and Levi, 2011; Gori and Facoetti, 2015; Grainger et al., 2016).

### Accentuations in biology

To conclude these sections, we suggest some biological implications about the way the principle of accentuation is used in nature by different organisms.

**Figure 14:**
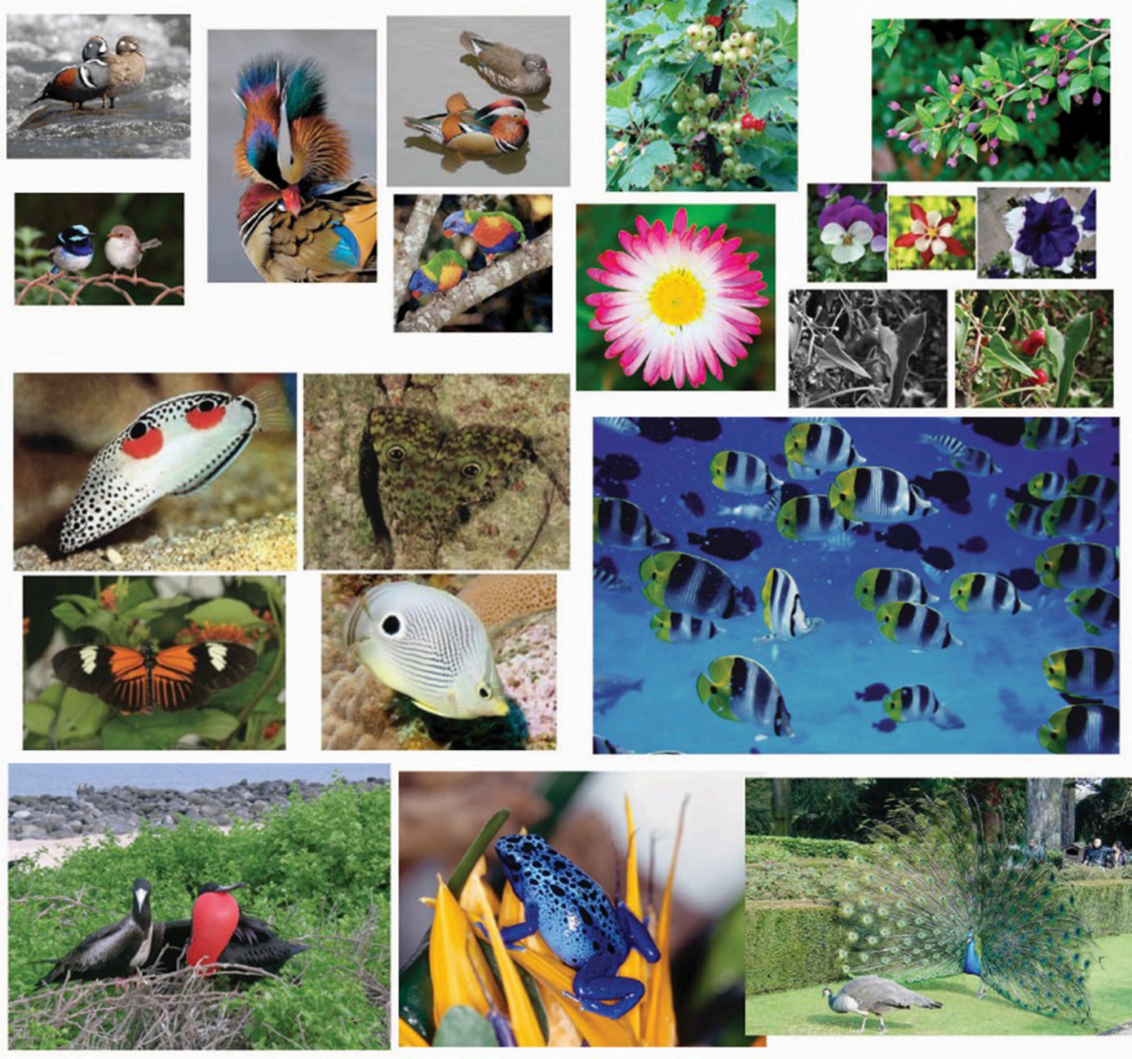
**Biological implications of accentuation and of coupling: Masking and unmasking effects in animals due to the principle of accentuation**.

The masking and unmasking effect due to similarity can be easily found in nature, for example in camouflages by mimesis and crypsis, according to which, animals otherwise visible, remain unnoticed by resembling to something else (birch, willow branches, dry leaf, etc.) or by blending with their environment. Both kinds of camouflages are only apparently in contradiction with the assumption of homogeneity, previously described. As a matter of fact, the whole unique object, based on similarity and homogeneity of its components, is not the animal but the environment. In other words, being the animal homogeneous with its environment, it becomes environment. However, to become environment the organism should incorporate elements by patterning and rephrasing the accents of their livery. This is the case of camouflage by crypsis, where the disruptive patterning is accomplished by some organisms by means of strong contrasting markings, like spots or stripes, to break up their own outlines. Typically, the high-contrast patches in a non-repetitive configuration provide camouflage by disrupting the recognizable shape or the orientation of the animal. Some examples are illustrated in Figure 14.

In short, disruptive camouflage (see Merilaita and Lind, 2006) is a way to confuse an individual organism with high contrasted colorations and markings, which disguise the whole shape and the shape of the parts of the body. These markings appear quite distinctive to prevent the predator from accurately identifying shape, size, and orientation (see Stevens et al., 2007). This is consistent with our previous results.

There is a specific kind of disruptive camouflage strongly related to the accentuation principle concerning false eyes (ocelli) and dots (diematic patterns) on the livery of some organisms and it demonstrates what can be called “deceiving camouflage by accentuation.” This camouflage, very similarly to the disruptive effect shown in Arnheim's quote of Figure 13, is aimed at confusing (changing the body organization, size, orientation, and motion direction, as shown in the fishes illustrated in Figure 14) and hiding the most vital and important parts of their body (e.g., the butterflies). By depending on the principle of accentuation, these kinds of disrupting and deceiving effects play different biological roles at the same time. Not only are they defense mechanisms but also they are crucial to startle or frighten potential predators. Besides they can also become source of sexual attraction (see the birds of Figure 14), can help to advertise the presence (see the flowers and the fruits of Figure 14) and can also elicit species identification/communication (see also Stevens et al., 2006; Stevens, 2007; Stevens and Merilaita, 2009; Troscianko et al., 2009; Stevens and Ruxton, 2012; Pinna and Reeves, 2015).

By rethinking of the differences between salience and accentuation, described in Section Accentuation vs. Salience and Coupling vs. Medium-Range Grouping, within the biological domain, the role of the accent can now be reconsidered in a more appropriate way. Its role is not only related to highlight or hide a specific component but also it is important to impart to the whole organism a special status, a special attribute that is the one of being accentuated. In greater details, accentuation not only conveys disrupting and deceiving camouflage, but it can also impart strength, power, sexual attraction, etc. In other terms, while salience is restricted to the specific component that can thus become more visible, accentuation spreads the accentuated attribute or element by filling the entire holder with its new properties. This specific and unique effect due to the accentuation is true, all the more reason, within the human domain, as described more in details in the next section. As a matter of fact, maquillage and other kinds of accentuation due to elements or objects “coupled” with humans (e.g., clothes, watches, shoes, cars, houses, etc.) impart to the owner special attributes or a special status which do not differ from those emerging on the base of accentuations observed on the livery, plumages, wings, bodies, or heads of all the kinds of organisms in nature. These results can be uniquely explained by the new key concepts of accentuation and coupling here introduced. Moreover, these results are far beyond the Gestalt theory of perceptual grouping and require the introduction of a new kind of perceptual organization that we have called “coupling,” a new concept that is ruled by the basic principle of accentuation. Further theoretical implications will also be discussed in the final section.

### Accentuation in humans

**Figure 15:**
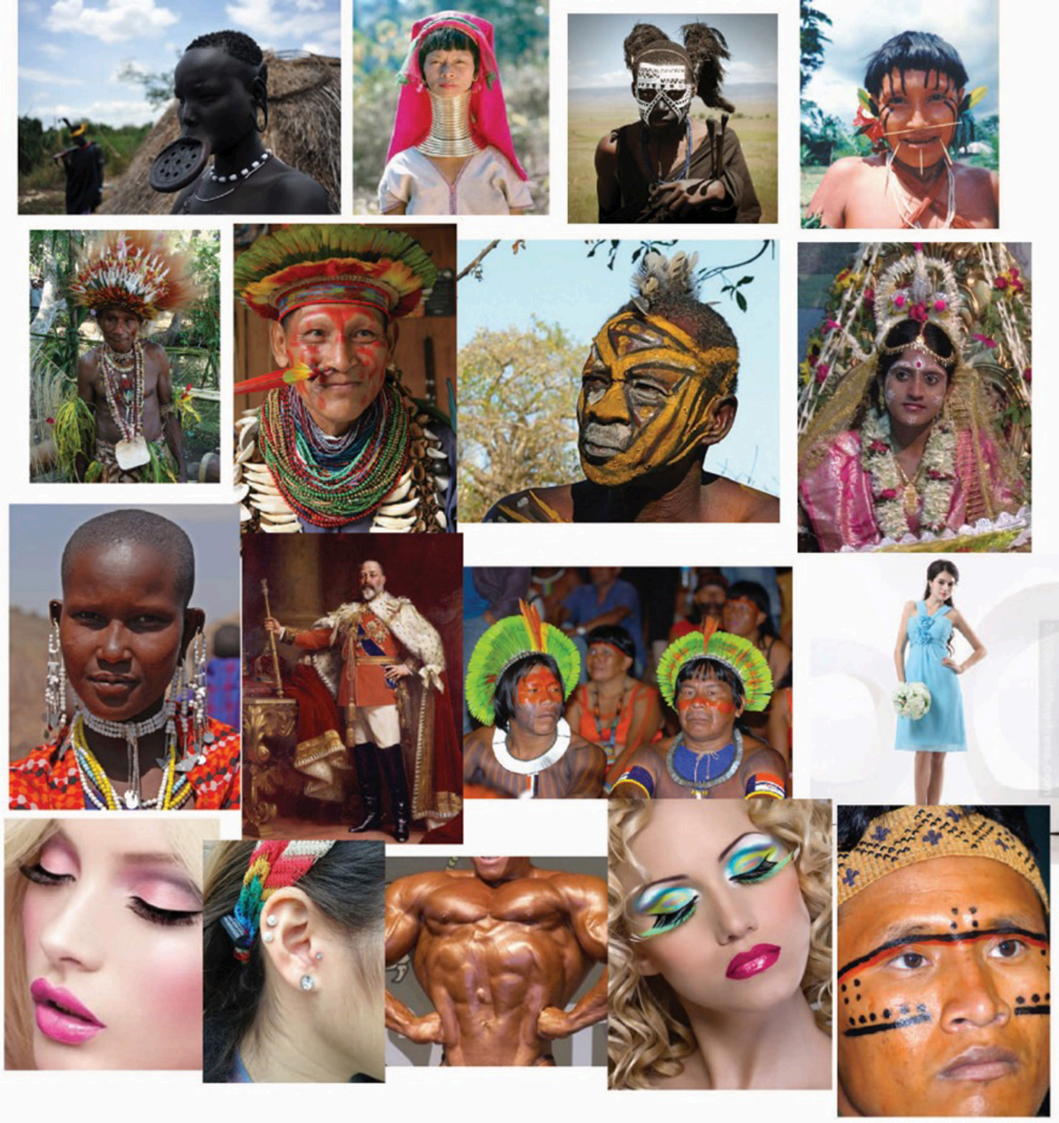
**The basic role of the principle of accentuation and coupling in different contexts and aspects of human life**.

Nature knows the complex language of accents and coupling. Everything around us is a swarm of accents of different kind and placed in different positions. Accents are needed to communicate, to camouflage, to confuse, to blur, to clarify, to interpret, to convey meaning. This complexity is well-represented in human beings (see Figure 15), e.g., in the way we show ourselves to others, in the way we dress, choose, and create clothes and invent fashion, but also in the way we use and invent design, in the way we change our body accentuating several parts and hiding some others, in the way we use the maquillage, in the existence of the maquillage itself.

In deceiving, by means of magic tricks and card tricks, the use of accentuation is essential and effective only if it hides or displaces the attention of the observer. A further remark on the role of attention related to the principle of accentuation can be stated in short as follows: it is the accent that attracts and creates a displacement of the attention and not the attention that creates the accent.

The principle of accentuation can also be used to highlight specific parts of the body. For example, it can be used to increase sexual attraction (see the examples included in Figure 15), to exhibit physical strength, to show a special social status (e.g., being the king, a warrior, a priest, etc.), but it is also a mean to establish personal identity (this is the need to accentuate a role inside a group, within a family, or an institution), and to manifest the fact of being the number one within a group (for example the Pope is the only priest who dresses in white as ruled by the dissimilar accentuation).

## Discussion

In the previous sections perceptual organization has been studied with the same spirit and phenomenological methods used by Gestalt psychologists. This was accomplished by investigating new conditions that cannot be explained in terms of classical grouping principles. They represent borderline stimuli that contradict the notion of grouping as aimed to account for how the elements in the visual field “go together” to form an integrated percept, namely, how they create larger wholes together with other elements. More generally, perceptual grouping represents the way through which our visual system builds integrated elements on the basis of the maximal homogeneity among the components of the stimulus pattern. Their homogeneity is ruled by the well-known general principles including similarity that is the main principle on which our study was focused.

Our results demonstrated the inconsistency and incompleteness of grouping, when considered as the only way of visual organization, and more particularly, the inconsistency and incompleteness of the principle of similarity. On the contrary, they suggested the basic role played by dissimilarity among elements that behaves like an accent or a visual emphasis within a whole. Accentuation derives from dissimilarity and states that, all else being equal, elements tend to group in the same oriented direction of the dissimilar element placed within or outside a whole set of continuous/homogeneous components.

The principle of accentuation was here demonstrated as imparting a directional structure to the elements within a whole object and to the whole object itself. More precisely, accentuation was used to highlight properties such as “sidedness”and “pointedness” belonging to sides and angles within squares presented rotated at 45°. Then, it was demonstrated that accentuation of the sidedness goes together with the perception of a rotated square, while accentuation of the pointedness goes with the perception of the diamond within the same stimulus.

The strength of the resulting phenomena here studied revealed the supremacy of dissimilarity in relation to similarity and the fact that it belongs to further organization dynamics that we called “coupling.” It was also shown that, while similarity induces homogeneity, dissimilarity creates discontinuities that spread in the entire surrounding homogeneous field of components. This entails that, whether, on one hand, similarity is one of the main principles of grouping defining “who is with who and who is against who,” on the other hand, dissimilarity is the source of a new kind of organization that couples and connects similarities and dissimilarities in a new way and at a different perceptual level.

Within the notion of coupling it is subsumed the idea of a reciprocal interaction and influence in between dissimilar elements. This is not expected on the basis of the dynamics of grouping. The two components of the couple are two and one at the same time: two, since they are not perceived as grouped in the same acceptation suggested by Gestalt psychologists; one, since they clearly influence each other in attributes like “sidedness” and “pointedness.”

It was also suggested that coupling allows binding of many dissimilar kinds of elements, which would otherwise take place in isolation if considered only on the basis of the principles of grouping. This can explain the complex bond created among objects of every kind in our visual world, not necessarily homogeneous as required by perceptual grouping.

It was also demonstrated that the principle of accentuation does not depend on visual attention and that, in biology, accentuation is very strongly related to disruptive camouflage as a way to confuse an individual organism with high contrasted markings and coloration, whose purpose is to disguise the whole shape and the shape of some parts of its body. Moreover, accentuation comes to startle or frighten potential predators, it is source of sexual attraction, it advertises the presence and elicits species identification/communication.

The principle of accentuation also plays a basic role in human beings and in all our lives. As a matter of fact, related to accentuation is the way we show ourselves to others, the way we dress, choose and create clothes and invent fashion, the way we change our body accentuating several parts of it and hiding some others, the way we use maquillage. The existence of maquillage itself is derived from the need to accentuate something with the purpose to increase sexual attraction, to exhibit physical strength and beauty, to show or hide social status (e.g., being the king, a warrior, a priest, etc.). Last but not least, accentuation plays a basic role also in making it easier or difficult to read and understand written words. The list, here closed, can continue *ad libitum*.

It is worthwhile to underline that visual accentuation can be considered as a principle in the sense of a distinct brain design that can be described similarly to every other kind of phenomenal regularity and homogeneity within the contest of laminar visual cortical circuits. For this purpose, the best candidates to explain the complexity of this principle and, more generally, the complexity of coupling are the FACADE neural models of 3-D vision and grouping (Grossberg, 1999, 2003) and the LAMINART (Grossberg and Raizada, 2000; Raizada and Grossberg, 2003; Grossberg and Swaminathan, 2004; Grossberg and Yazdanbakhsh, 2005), both aimed to explain how complementary cortical boundary and surface representations interact with spatial attention to generate conscious percepts of grouping and 3-D form. Since they involve the spatial attention, in the light of the feedback derived from our phenomenal results that deny the role of attention, we suggest that the two models can receive due changes to better fit our outcomes.

## Ethic statement

All subjects gave written consent in accordance with the declaration of Helsinki.

## Author contributions

BP, DP, KD, all authors have given a substantial contribution in all the four areas listed in the criteria section. Of course the corresponding author BP gave more in term of critical experience, drafting, and designing the work, as well as bringing an important intellectual contribution. KD and DP have given their contribution in acquisition and analysis of data for example, without neglecting to play an active part in all the other areas collaborating with the corresponding author in every stage of the work. However, everybody was happy and agreed to all the solutions taken and to the final version of the work.

### Conflict of interest statement

The authors declare that the research was conducted in the absence of any commercial or financial relationships that could be construed as a potential conflict of interest. The reviewer AY and handling Editor declared their shared affiliation, and the handling Editor states that the process nevertheless met the standards of a fair and objective review.
